# Human Galectin-9 Is a Potent Mediator of HIV Transcription and Reactivation

**DOI:** 10.1371/journal.ppat.1005677

**Published:** 2016-06-02

**Authors:** Mohamed Abdel-Mohsen, Leonard Chavez, Ravi Tandon, Glen M. Chew, Xutao Deng, Ali Danesh, Sheila Keating, Marion Lanteri, Michael L. Samuels, Rebecca Hoh, Jonah B. Sacha, Philip J. Norris, Toshiro Niki, Cecilia M. Shikuma, Mitsuomi Hirashima, Steven G. Deeks, Lishomwa C. Ndhlovu, Satish K. Pillai

**Affiliations:** 1 Blood Systems Research Institute, San Francisco, California, United States of America; 2 University of California, San Francisco, California, United States of America; 3 School of Biotechnology, Jawaharlal Nehru University, New Delhi, India; 4 Hawaii Center for AIDS, John A. Burns School of Medicine, University of Hawaii, Honolulu, Hawaii, United States of America; 5 RainDance Technologies, Inc., Billerica, Massachusetts, United States of America; 6 Vaccine & Gene Therapy Institute, Oregon Health & Science University, Portland, Oregon, United States of America; 7 Oregon National Primate Research Center, Oregon Health & Science University, Portland, Oregon, United States of America; 8 GalPharma Co., Ltd., Takamatsu-shi, Kagawa, Japan; 9 Department of Immunology and Immunopathology, Kagawa University, Kagawa, Japan; Emory University, UNITED STATES

## Abstract

Identifying host immune determinants governing HIV transcription, latency and infectivity *in vivo* is critical to developing an HIV cure. Based on our recent finding that the host factor p21 regulates HIV transcription during antiretroviral therapy (ART), and published data demonstrating that the human carbohydrate-binding immunomodulatory protein galectin-9 regulates p21, we hypothesized that galectin-9 modulates HIV transcription. We report that the administration of a recombinant, stable form of galectin-9 (rGal-9) potently reverses HIV latency *in vitro* in the J-Lat HIV latency model. Furthermore, rGal-9 reverses HIV latency *ex vivo* in primary CD4+ T cells from HIV-infected, ART-suppressed individuals (p = 0.002), more potently than vorinostat (p = 0.02). rGal-9 co-administration with the latency reversal agent "JQ1", a bromodomain inhibitor, exhibits synergistic activity (p<0.05). rGal-9 signals through N-linked oligosaccharides and O-linked hexasaccharides on the T cell surface, modulating the gene expression levels of key transcription initiation, promoter proximal-pausing, and chromatin remodeling factors that regulate HIV latency. Beyond latent viral reactivation, rGal-9 induces robust expression of the host antiviral deaminase APOBEC3G *in vitro* and *ex vivo* (FDR<0.006) and significantly reduces infectivity of progeny virus, decreasing the probability that the HIV reservoir will be replenished when latency is reversed therapeutically. Lastly, endogenous levels of soluble galectin-9 in the plasma of 72 HIV-infected ART-suppressed individuals were associated with levels of HIV RNA in CD4+ T cells (p<0.02) and with the quantity and binding avidity of circulating anti-HIV antibodies (p<0.009), suggesting a role of galectin-9 in regulating HIV transcription and viral production *in vivo* during therapy. Our data suggest that galectin-9 and the host glycosylation machinery should be explored as foundations for novel HIV cure strategies.

## Author Summary

While antiretroviral therapy (ART) has significantly decreased the morbidity and mortality associated with HIV infection, a cure is not achieved due to the persistence of HIV latently-infected cells during treatment. Identifying the principal host immune determinants governing HIV transcription, latency, and infectivity *in vivo* will be a critical step in developing an effective curative strategy for HIV infection. In this study, we demonstrate that the human immunomodulatory carbohydrate-binding protein galectin-9 is a determinant of HIV latency in HIV-infected individuals on suppressive ART. Administration of galectin-9 potently reactivates latent HIV in CD4+ T cells *ex vivo*, by signaling through specific glycans on the cell surface to modulate the gene expression levels of key host factors that regulate HIV transcription. Furthermore, galectin-9 induces the host APOBEC3 proteins which lethally mutagenize the HIV genome, attenuating viral infectivity. Our findings reveal a novel biological function of human galectin-9, and demonstrate that host glycans on the surface of infected cells mediate signals that define the transcriptional state and infectivity of HIV. Our findings suggest that galectin-9 and the host glycosylation machinery may be exploited to eradicate the latent HIV reservoir.

## Introduction

Antiretroviral therapy (ART) has demonstrated efficacy and durability in suppressing HIV replication in infected individuals. However, ART does not achieve viral eradication due to the persistence of latently infected long-lived cells [1,2]. Several recent studies demonstrating continued morbidity during suppressive ART have created profound interest in developing a cure for HIV infection. The elimination of the latent reservoir is critical to achieving HIV eradication, as demonstrated by resurgence of virus post ART-cessation [3,4]. Alternatively, a “functional cure” involving control of virus to undetectable levels in the absence of complete eradication may be established to minimize ART-associated morbidity and enable ART-independent suppression of HIV to clinically undetectable levels, as demonstrated in recent clinical studies [5]. Identifying host determinants governing HIV transcription, latency, and infectivity *in vivo* will be a critical step in developing both of these curative modalities for HIV infection.

The “shock and kill” strategy is currently one of the most widely discussed approaches to eliminate the viral reservoir [6]. In this approach, drugs are administered to reverse HIV latency and induce viral production, ultimately resulting in the death of infected cells by direct viral cytopathic effects or immune-mediated clearance. Latency reversing agents (LRAs) are administered during suppressive ART, thereby preventing reactivated virus from replenishing the reservoir through infection of new cells. Clinical trials involving LRAs such as romidepsin, vorinostat, disulfiram, and panobinostat have failed to demonstrate significant reduction in reservoir size, although transient elevation in plasma viral RNA has been observed [7–13]. Accordingly, *ex vivo* experiments have revealed that the majority of existing LRAs exert weak effects on HIV transcription and reactivation [14]. The future success of shock and kill will depend on our capacity to design or identify highly efficacious LRAs and/or adjuvant therapies to boost the reactivation potential of existing LRAs.

Based on our recent finding that the p21 (CDKN1A) host restriction factor and cell cycle regulator [15,16] modulates HIV transcription in ART-suppressed HIV-infected individuals [17], and reports suggesting that the human lectin galectin-9 (Gal-9) regulates p21 expression [18–20], we pursued the hypothesis that Gal-9 modulates HIV transcription and latency. The galectin family of animal lectins consists of a group of glycan-binding proteins characterized by conserved carbohydrate recognition domains (CRDs), defined by shared consensus amino acid sequences which confer specific binding to β-galactoside-containing glycoconjugate proteins [21]. Galectins are ubiquitously expressed throughout the animal kingdom, from lower organisms, such as nematodes and sponges, to higher mammalian species, including humans [22]. Fifteen members of the mammalian galectin family have been identified to date [23]. Gal-9 has been recently recognized to play an important role in several diseases including HIV infection through regulation of both adaptive and innate defense mechanisms [24–26]. Recombinant Gal-9 (rGal-9) has been used successfully and safely as a therapy in a number of mouse disease models including graft versus host disease [27], rheumatoid arthritis [28], asthma [29], leukemia [30], and colon cancer [31].

## Results

### rGal-9 potently reverses HIV latency *in vitro*

**Fig 1:**
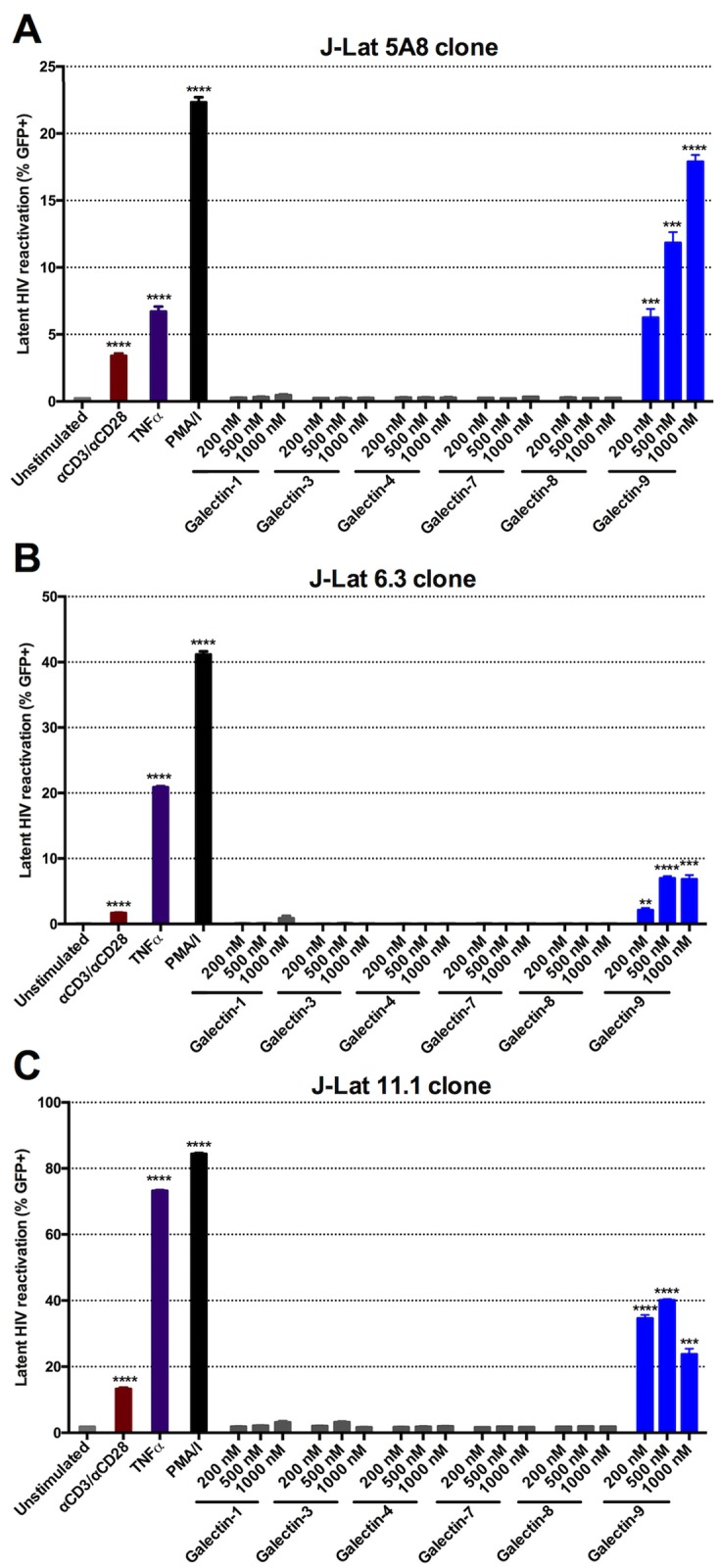
rGal-9 is a potent mediator of HIV transcription *in vitro*. *in vitro* HIV reactivation in the J-Lat latency model **(A)** 5A8 clone, **(B)** 6.3 clone, and **(C)** 11.1 clone by varying doses of rGal-9 and other galectins (-1, -3, -4, -7, -8, and -9) after 24 hours of stimulation. αCD3 /αCD28 antibodies conjugated to beads, PMA/ionomycin (16 nM/500 nM), and TNFα (10 ng/ml) were used as positive controls. J-Lat cells were analyzed by flow cytometry to assess HIV-encoded GFP expression. Mean ± SEM is displayed, and statistical comparisons were performed using two-tailed unpaired t tests. * = p<0.05; ** = p<0.01, *** = p<0.001, and **** = p<0.0001.

We recently demonstrated that the expression levels of particular cell-intrinsic immune factors are associated with levels of cell-associated HIV RNA in the setting of ART [17]. Based on reports demonstrating that Gal-9 regulates the expression of these key cell-intrinsic immune mechanisms [18–20], we hypothesized that manipulation of Gal-9 may induce transcription of latent HIV. We initially assessed the ability of a biologically active and stable form of rGal-9 [32,33] to modulate HIV transcription and reactivate latent HIV in the established “J-Lat” model of HIV latency. J-Lat cells harbor a latent, transcriptionally competent HIV provirus that encodes green fluorescent protein (GFP) as an indicator of reactivation [34,35]. We performed dose-response experiments by stimulating J-Lat 5A8, 6.3 and 11.1 clones [36] with rGal-9 for 24 hours. Our data revealed that rGal-9 reactivated latently-infected J-Lat cells in a dose-dependent manner (up to 17.9% in the 5A8 clone, up to 6.96% in the 6.3 clone, and up to 40.1% in the 11.1 clone), and with potency greater than that seen with αCD3/αCD28 stimulation (Fig 1A–1C). When we assessed the ability of other galectins (galectins-1, 3, 4, 7, 8) to reverse HIV latency, none of the other galectins (including those structurally similar to galectin-9, such as the other tandem-repeat type galectins, galectin-4 and galectin-8) exhibited any capacity to reactivate latent HIV infection at the same concentrations used for rGal-9, suggesting that HIV latency reversal is a unique feature of Gal-9 amongst members of the galectin family (Fig 1A–1C). Limiting duration of exposure of the J-Lat 5A8 cell to 1000 nM of rGal-9 for 6 hours achieved ~67% of the activity observed after continuous 24 hours of treatment, while pulse treatment for only one hour achieved equivalent reactivation to αCD3/αCD28 stimulation (S1 Fig). In addition, we examined the effects of rGal-9 treatment on the expression of the HIV p24 and Vif proteins in J-Lat 5A8 cell lysates. Our data demonstrate that rGal-9 induces the expression of HIV viral proteins (S2 Fig).

### rGal-9 potently reverses HIV latency *ex vivo*

**Fig 2:**
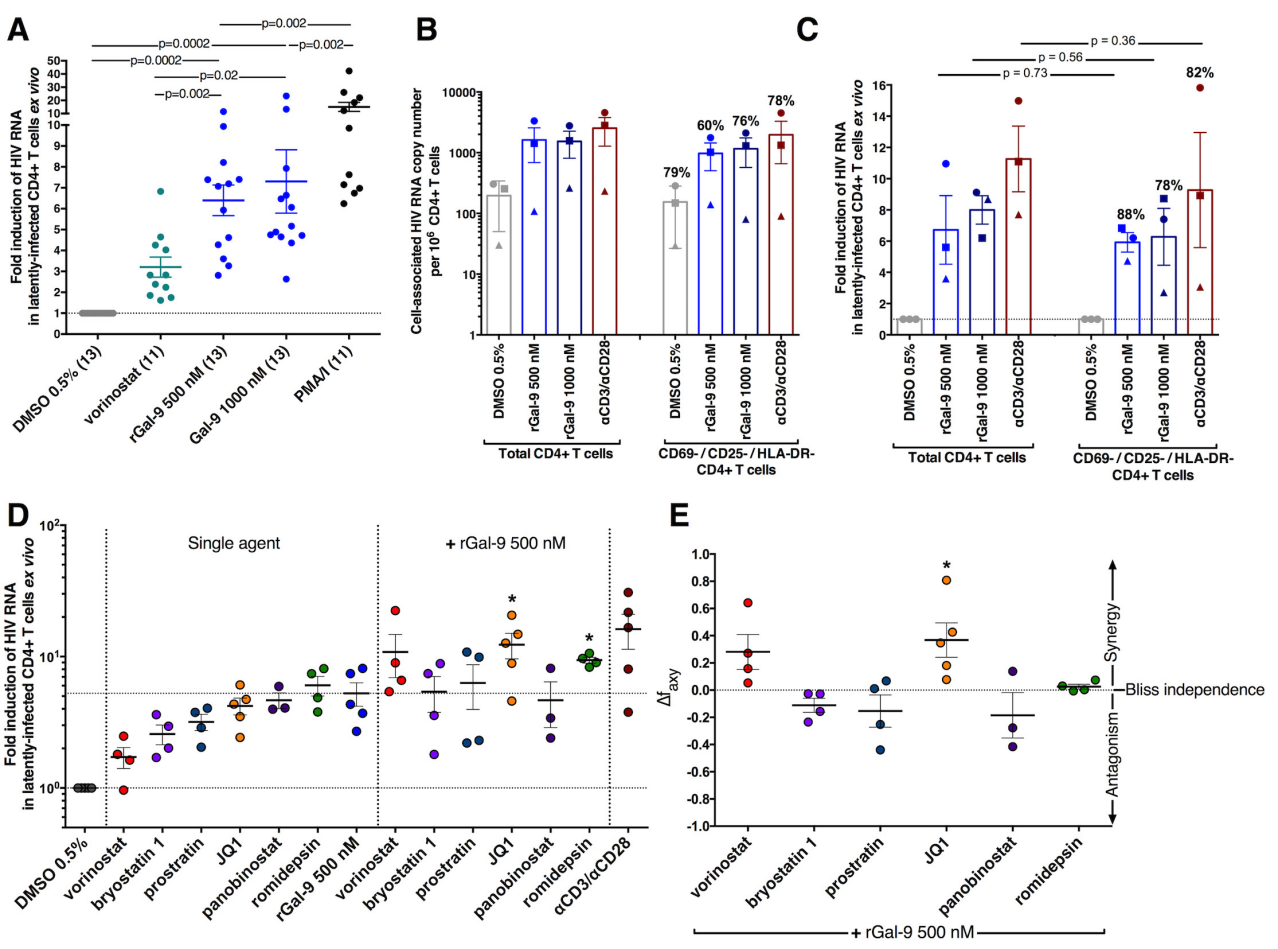
rGal-9 is a potent mediator of HIV transcription *ex vivo and synergizes with JQ1 in reactivating latent HIV*. (**A**) Treatment of CD4+ T cells isolated from ART-suppressed HIV-infected individuals with DMSO 0.5% (negative control), PMA/ionomycin (2 nM / 500 nM), vorinostat (1μM), or varying concentrations of rGal-9 (500 nM and 1000 nM) for 24 hours. Fold increase in cell-associated HIV RNA was determined relative to the corresponding DMSO-treated control for each individual time point. Mean ± SEM is displayed, and statistical comparisons between rGal-9 and other treatments were performed using two-tailed paired Wilcoxon signed-rank tests. **(B-C)** CD4+ T cells were isolated from PBMCs of three HIV-infected ART-suppressed individuals using negative selection. Resting CD4+ T cells were further enriched through depletion of cells expressing CD69, CD25, or HLA-DR surface markers from half of the isolated CD4+ T cells. The remaining half was processed through the exact enrichment procedure, except PBS was added instead of the depleting antibodies. Both cell populations were treated with 0.5% DMSO (negative control), 500 nM rGal-9, 1000 nM rGal-9 or αCD3/αCD28-conjugated beads. Induction of cell-associated HIV RNA was measured 24 hours post-treatment using RT-qPCR. Each individual is represented with a different symbol. Mean ± SEM is displayed, and statistical comparisons were performed using two-tailed paired t tests. Percentages reported reflect average values measured in the CD69- / CD25- / HLA-DR- CD4+ T cells with respect to values observed in total CD4+ T cells. **(D)** CD4+ T cells from HIV-infected ART-suppressed individuals were treated with 500 nM of rGal-9, 1 μM vorinostat, 40 nM romidepsin, 10 nM bryostatin, 300 nM prostratin, 1 μM JQ1, or 30 nM panobinostat alone or in combination with 500 nM of rGal-9 for 24 hours, and fold induction of cell-associated HIV RNA was determined using quantitative real-time PCR. * = p<0.05 compared with rGal-9 500 nM treatment alone. **(E)** The Bliss independence model was utilized for calculation of synergy for drug combinations. Δf_axy_ = 0 signifies a pure additive effect. Δf_axy_>0 signifies synergy, while Δf_axy_<0 signifies antagonism. Statistical significance was calculated using a two-tailed paired t-test comparing predicted and observed drug combination effects. * = p < 0.05.

We assessed the ability of rGal-9 to reactivate latent HIV in isolated CD4+ T cells derived from 13 HIV-infected individuals on suppressive ART. CD4+ T cells were treated with either DMSO 0.5% as negative control, rGal-9 at two different concentrations (500 nM and 1000 nM), PMA/ionomycin (2 nM, 500 nM), or vorinostat (1μM) for 24 hours. 500nM and 1000 nM of rGal-9 induced a mean 6.4-fold and 7.3-fold increase in intracellular HIV RNA levels after 24 hours, respectively, as compared to DMSO negative control (p = 0.002). Induction was significantly higher than vorinostat (p = 0.02, 3.2 fold) (Fig 2A). Depleting CD4+ T cells expressing CD69, CD25, HLA-DR surface activation markers did not significantly affect rGal-9-mediated latent HIV reactivation *ex vivo* in CD4+ T cells, determined in a representative subset of three HIV-infected ART-suppressed individuals (Fig 2B and 2C). We additionally investigated the effects of limited exposure *ex vivo* in the same subset of three HIV-infected ART-suppressed individuals. Our data demonstrate that six hours of exposure to 1000 nm of rGal-9 induces an average of 59.2% of the viral reactivation observed after 24 hours of continuous exposure *ex vivo* (S3 Fig).

We next explored synergy between rGal-9 and established latency reversal agents. In five HIV-infected ART-suppressed individuals, CD4+ T cells were treated with 500 nM of rGal-9, 1 μM vorinostat [7,10,11,14], 40 nM romidepsin [14,37], 10 nM bryostatin [14,38,39], 300 nM prostratin [14,40–42], 1 μM JQ1 [14,43–45], or 30 nM panobinostat [14,46,47] alone or in combination with 500 nM of rGal-9, in addition to αCD3 + αCD28-conjugated beads (Dynal, at 1:1 bead:cell ratio) as positive control. Fold induction of cell-associated HIV RNA was determined using quantitative real-time PCR 24 hours after treatment. Based on the Bliss independence model for the quantitative analysis of synergy [14,43,48], we determined that rGal-9 co-administration with the latency reversal agent "JQ1", a bromodomain inhibitor, exhibits synergistic activity (p<0.05) (Fig 2D and 2E). In addition, we evaluated the effects of rGal-9 on the viability of freshly obtained primary CD4+ T cells from multiple HIV-infected ART-suppressed individuals. Our experiment demonstrated that rGal-9 at high dosages (500 nM and 1000 nM) results in 15–20% reduction in cell viability, which is comparable to or better than existing LRAs such as romidepsin [37] (S4 Fig).

### rGal-9 reverses HIV latency in a glycan-dependent manner

**Fig 3:**
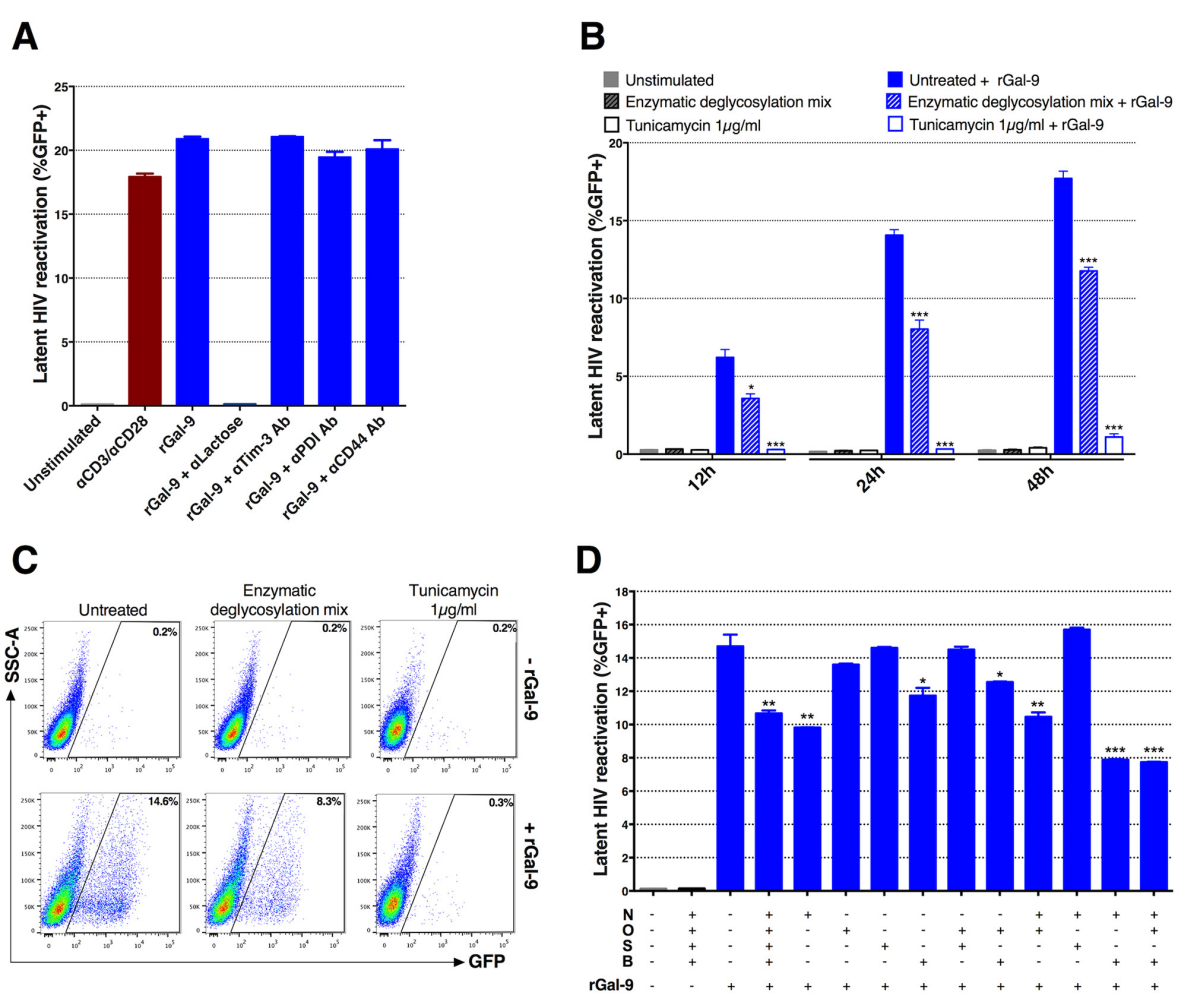
rGal-9 induces HIV transcription and reactivation in a glycan-dependent manner. (**A**) Effects of anti-Tim-3 antibody, anti-CD44 antibody, or anti-PDI antibody administration on rGal-9-mediated reactivation of HIV in J-Lat 5A8 cells. Antibodies were added 30 minutes prior to administration of 200 nM rGal-9. α-lactose (30 mM) was used as a positive control. (**B**, **C**) Treatment of J-Lat 5A8 cells with either 1 μg/ml tunicamycin, or with an enzymatic deglycosylation mix for 24 hours prior to rGal-9 stimulation. J-Lat cells were analyzed by flow cytometry to assess HIV-encoded GFP expression. Statistical comparisons were performed using two-tailed Mann-Whitney tests. (**D**) Effects of deglycosylation enzyme combinations on rGal-9-mediated HIV latency reversal in J-Lat 5A8 cells. N = PNGase F (Elizabethkingia miricola); O = O-Glycosidase (recombinant from Streptococcus pneumonia); S = α-(2→3,6,8,9)-Neuraminidase (recombinant from Arthrobacter ureafaciens); B = β(1→4)-Galactosidase (recombinant from Streptococcus pneumonia) + β-N-Acetylglucosaminidase (recombinant from Streptococcus pneumonia). Mean ± SEM is displayed, and statistical comparisons were performed using two-tailed unpaired t tests. * = p<0.05; ** = p<0.01, *** = p<0.001, and **** = p<0.0001.

Published data have identified three T cell surface glycoproteins, T cell immunoglobulin and mucin protein-3 (Tim-3) [49], protein disulfide isomerase (PDI) [50], and CD44 [51], as receptors for Gal-9. Addition of anti-Tim-3 antibody, anti-PDI antibody, or anti-CD44 antibody prior to the addition of rGal-9 did not reduce rGal-9-mediated reactivation of latent HIV in 5A8 cells. α-lactose (30 mM), an established neutralizer of Gal-9 activity [24], completely inhibited rGal-9-mediated reactivation of latent HIV (Fig 3A), while other saccharide derivatives (glucose and galactose at 30 mM) did not affect rGal-9 capacity for latency reversal (S5 Fig). Based on the known carbohydrate-binding nature of lectins, we investigated the requirement for complex N- and O-glycans in rGal-9-mediated HIV latency reversal. J-Lat 5A8 cells were treated for 24 hours with either tunicamycin or an enzymatic deglycosylation mix prior to rGal-9 stimulation. Tunicamycin is an antibiotic that chemically blocks N-glycosylation of newly synthesized proteins at asparagine residues, and the deglycosylation mix enzymatically removes all N-linked and O-linked carbohydrates from glycoproteins. rGal-9-mediated HIV latency reactivation (%GFP+) was reduced from 14.1±0.6% to 0.3±0.03% (mean±SD) when glycan synthesis was chemically inhibited by tunicamycin, or to 8.0±1.0% with enzymatic removal of cell surface glycans after 24 hours (Fig 3B and 3C). Conversely, the effects of PMA/ionomycin on HIV latency reversal were enhanced by deglycosylation, while the activity of TNFα was unaffected (S6 Fig).

The enzymatic deglycosylation mix contains several enzymes (PNGase F, O-Glycosidase, α-(2→3,6,8,9)-Neuraminidase, β(1→4)-Galactosidase, and β-N-Acetylglucosaminidase). We tested the ability of each enzyme individually or in combination to reduce the capacity of rGal-9 to reverse HIV latency. A combination of PNGase F, β(1→4)-Galactosidase, and β-N-Acetylglucosaminidase demonstrated the most potent reduction of rGal-9 mediated HIV reactivation as compared to non-deglycosylated cells (14.7±1.2% to 7.9±0.1%) (Fig 3D). Thus, N-linked oligosaccharides and the less common, but widely distributed O-linked hexasaccharide structures (β(1→4)-linked galactose and β(1→6)-linked N-acetylglucosamine) are essential ligands in rGal-9-mediated latency reversal. These data suggest that the ability of rGal-9 to reverse HIV latency depends on binding to a preferred set of glycan structures on the T cell surface membrane to transduce intracellular signals driving HIV transcription.

### rGal-9 modulates the gene expression of key transcription initiation, chromatin remodeling and promoter-proximal pausing factors that regulate HIV transcription

**Fig 4:**
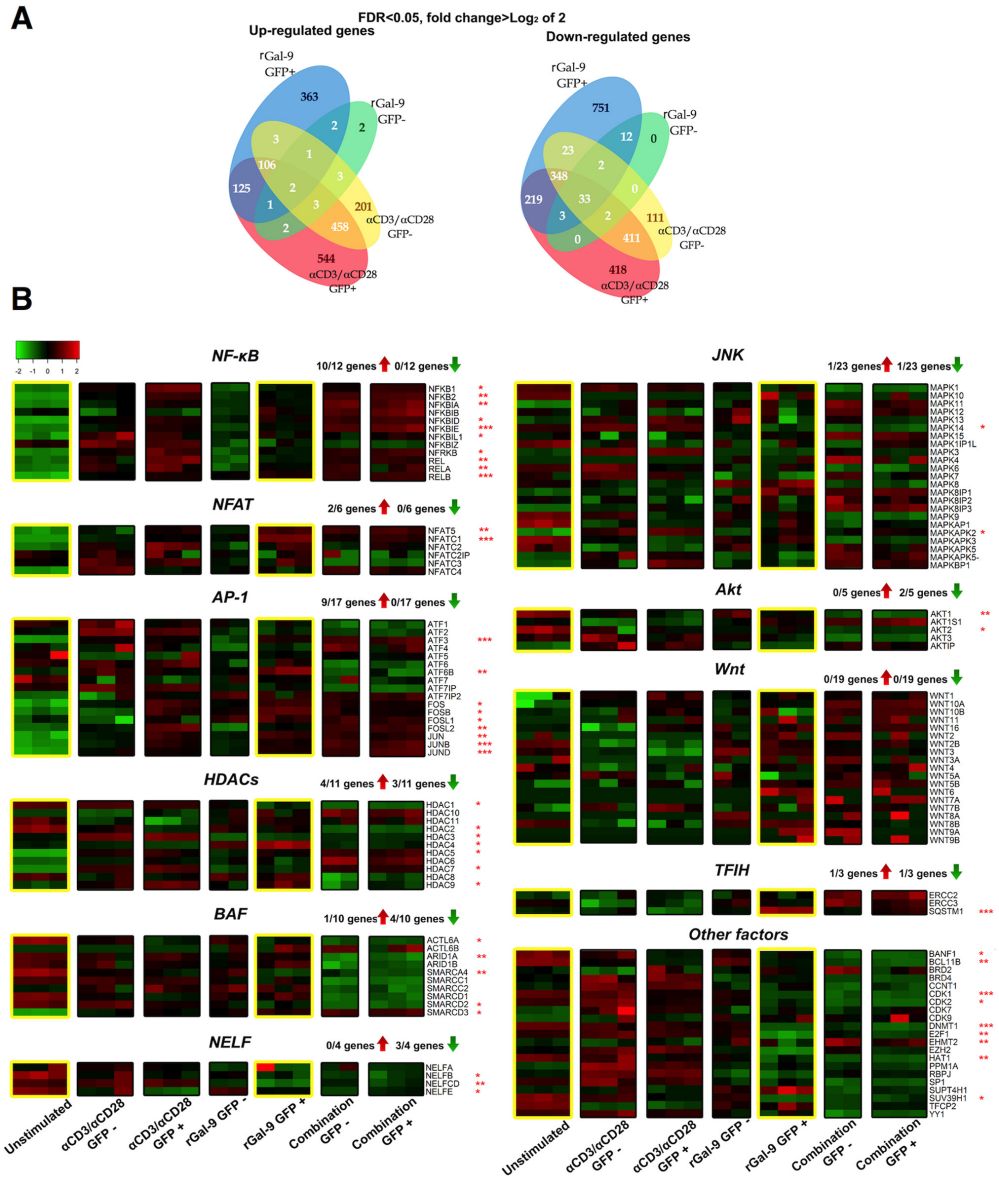
rGal-9 modulates the expression of genes involved in several signaling pathways associated with HIV latency. (**A**) Venn diagram showing the number of genes modulated by >2 fold with FDR<0.05 in sorted GFP-positive and GFP-negative cells containing reactivated (transcriptionally active) HIV proviruses, and latent (transcriptionally inactive) proviruses, respectively, after either rGal-9 treatment or αCD3/αCD28 stimulation. (**B**) Heat maps describing effects of rGal-9 treatment on host gene expression, organized by signaling pathways. All statistical comparisons were performed using t tests, and p values were adjusted for multiple comparisons using false discovery rate. Asterisks indicate >2-fold, statistically significant differences in gene expression between r-Gal9-treated, GFP+ cells and unstimulated control, as follows: * = FDR<0.05; ** = FDR<0.01, and *** = FDR<0.001.

We implemented RNA-sequencing (RNA-seq) to identify a host gene expression signature associated with rGal-9-mediated HIV latency reversal. We examined sorted GFP-positive and GFP-negative cells containing reactivated (transcriptionally active) HIV proviruses and latent (transcriptionally inactive) proviruses, respectively, after stimulation with rGal-9, αCD3/αCD28, or a combination of both agents. Cells with transcriptionally active proviruses (GFP+) as a result of rGal-9 stimulation exhibited a distinct gene expression pattern, compared to cells with transcriptionally active proviruses as a result of αCD3/αCD28 stimulation (Fig 4A, S7 Fig). We determined that rGal-9 significantly modulates the expression of genes involved in several established signaling pathways that are known to play important roles in maintaining HIV latency (FDR<0.05) (Fig 4B). rGal-9 induces the gene expression of several HIV proviral transcription initiation factors including the nuclear factor kappa-light-chain-enhancer of activated B cells (NFkB), AP-1 nuclear complex, and the calcium-dependent activator of HIV transcription initiation (NFAT). In addition, rGal-9 inhibits the gene expression of several chromatin modification and remodeling factors, including histone deacetylase 1, 2, and 3, EZH2, SUV39H1, DNMT1, BAF complex, and BCL11B. Lastly, rGal-9 inhibits the gene expression of the promoter-proximal pausing factors belonging to the NELF family.

### rGal-9 partially activates primary CD4+ T cells and induces naïve CD4+ T cell proliferation

**Fig 5:**
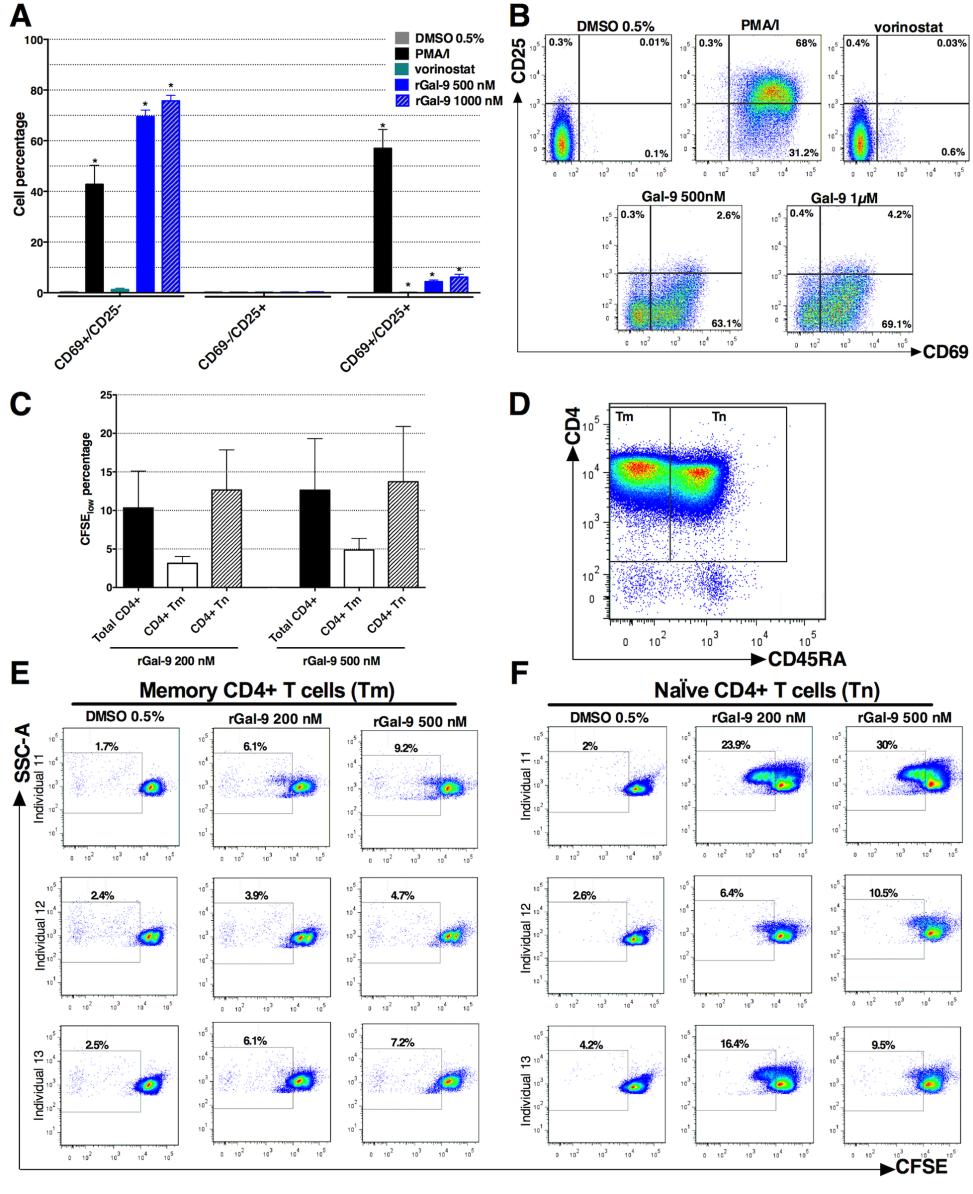
rGal-9 partially activates primary CD4+ T cells and induces proliferation primarily in naïve CD4+ T cells. (**A**, **B**) Effects of rGal-9 stimulation on the cell surface expression of CD69 and CD25 activation markers on CD4+ T cells isolated from six ART-suppressed individuals. Mean ± SEM is displayed. Asterisks represent statistically significant differences as compared to DMSO control (p < 0.05, two-tailed Wilcoxon signed-rank test). (**C**) Effects of rGal-9 stimulation on the proliferation of CD4+ T cells isolated from three ART-suppressed individuals. Primary CD4+ T cells were stained with CFSE and cultured for 5 days, stained with CD4 and CD45RA monoclonal antibodies, and proliferation was quantified as the percentage of CFSE_low_ cells on CD4+ CD45RA+ (Naïve, Tn) or CD4+ CD45RA- (Memory, Tm) T cells. Mean ± SEM is displayed. (**D**) Example of the flow cytometry gating strategy. (**E**, **F**) Effects of rGal-9 on proliferation of (**E)**, memory CD4+ T cells, and (**F)**, naïve CD4+ T cells.

In addition to evaluating the effects of rGal-9 on latent HIV reactivation, we sought to determine the effects of rGal-9 on primary CD4+ T cell phenotype, focusing on CD4+ T cell activation, proliferation and apoptosis. 500 nM and 1000 nM of rGal-9 induced surface expression of the CD69 activation marker (73.9%, 81.8%, respectively), and marginally induced CD25 (4.6%, 6.4%, respectively) (Fig 5A and 5B) on the surface of isolated CD4+ T cells from six HIV-infected ART-suppressed individuals. 200 nM and 500 nM of rGal-9 resulted in proliferation of primary CD4+ T cells isolated from three HIV-infected ART-suppressed individuals after five days of culture (10.3%, 12.6%, respectively) (Fig 5C), as measured by CFSE staining. rGal-9 selectively induced proliferation of naïve CD4+ T cells (CD4+ CD45RA+) (12.6%, 13.7%, respectively) rather than memory CD4+ T cells (CD4+ CD45RA-) (3.1%, 4.9%, respectively) (Fig 5E and 5F). In accordance with our *ex vivo* phenotypic data, we observed that rGal-9 modulates the expression of a subset of genes associated with regulation of T cell activation and proliferation *in vitro* (S8A–S8C Fig), and up-regulates multiple genes responsible for T cell apoptosis (S8E Fig).

### rGal-9 induces the expression of several anti-HIV host restriction factors including APOBEC3G

**Fig 6:**
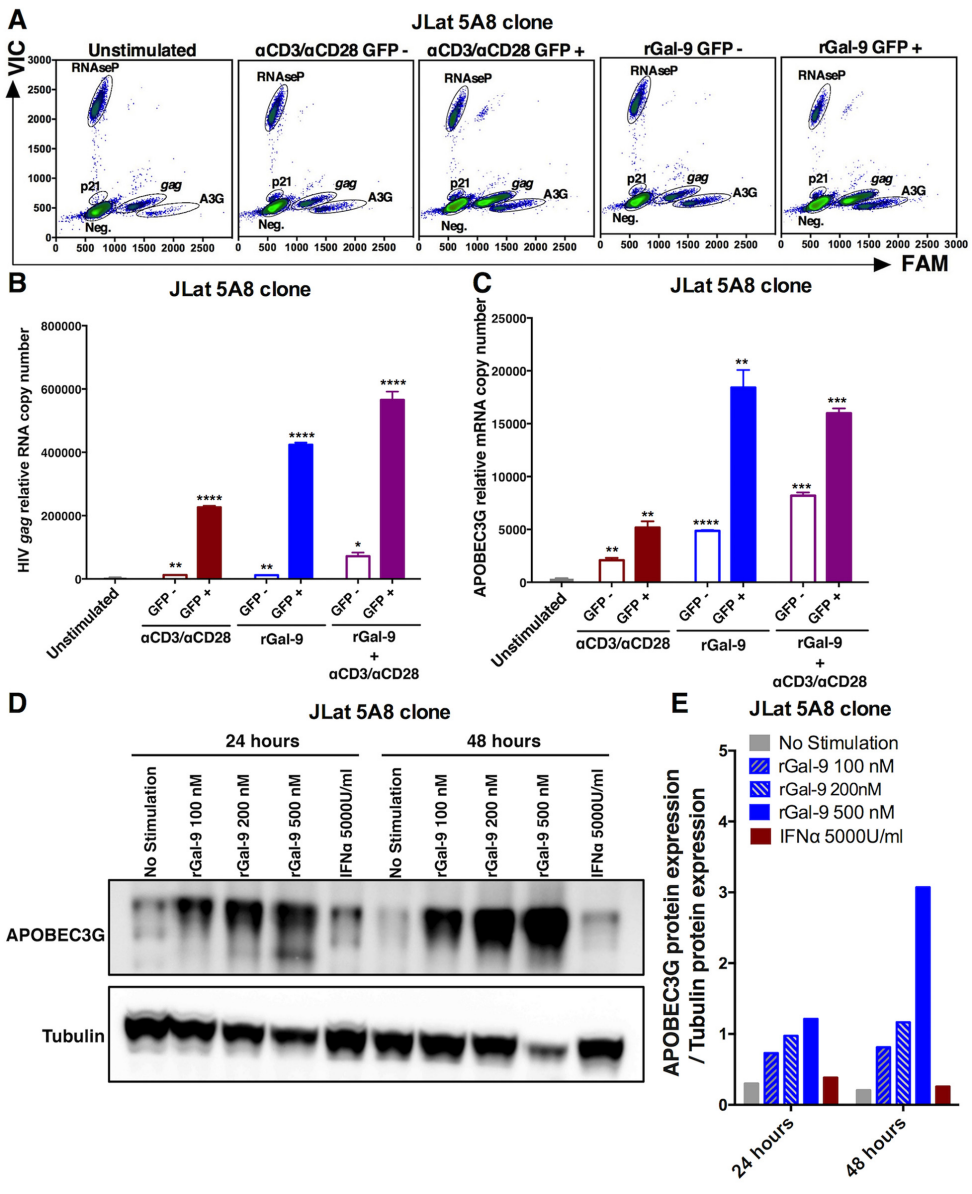
rGal-9 induces the expression of the APOBEC3G anti-HIV host restriction factor *in vitro*. (**A–C**) Digital droplet PCR gene expression profiling quantifying HIV *gag*, host APOBEC3G, p21, and RNAseP (housekeeping control) mRNA in J-Lat 5A8 cells sorted into GFP-positive and GFP-negative populations after either rGal-9 treatment, αCD3/αCD28 stimulation, or a combination of both. Mean ± SEM is displayed, and statistical comparisons against the unstimulated control were performed using two-tailed unpaired t tests. * = p<0.05; ** = p<0.01, *** = p<0.001, and **** = p<0.0001. **(D-E)** APOBEC3G protein expression in J-Lat 5A8 cells treated with varying concentrations of rGal-9 (100 nM, 200n M, and 500 nM) or interferon-α (5000 units/ml), as determined by western blot. Immunoblotting bands were quantified with ImageJ software. The quantified APOBEC3G protein expression levels were normalized to corresponding Tubulin protein levels to account for variation in loading.

**Fig 7:**
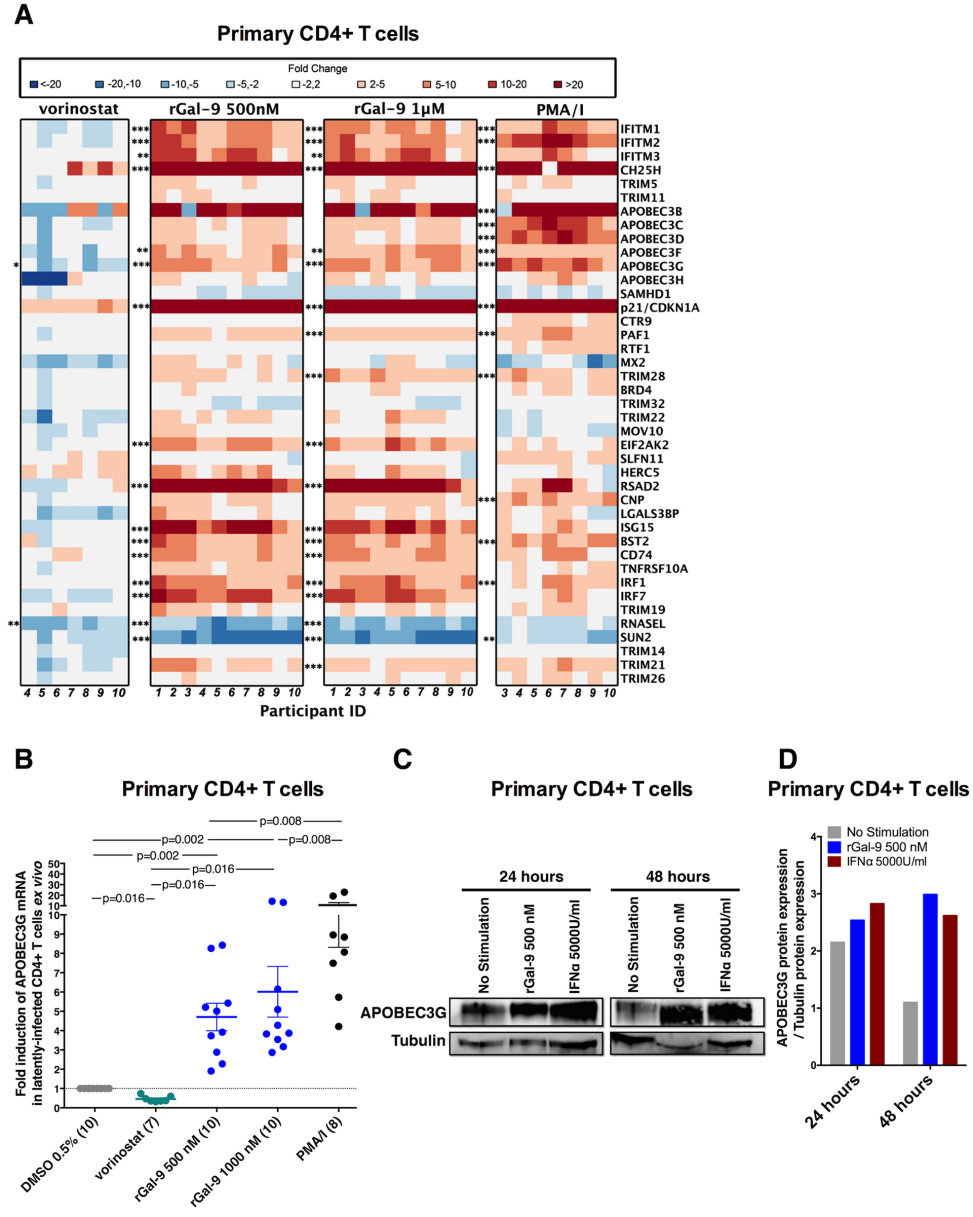
rGal-9 induces the expression of several anti-HIV host restriction factors including APOBEC3G *ex vivo*. (**A**) Heat map representing expression levels of host restriction factors in CD4+ T cells isolated from ART-suppressed individuals, after treatment with either 0.5% DMSO as negative control, 500 nM rGal-9, 1000 nM rGal-9, 1μM vorinostat, or a combination of PMA (2 nM) and Ionomycin (0.5 μM). Heat colors indicate fold modulation compared to the DMSO control. Red indicates induction of expression, and blue indicates reduction of expression. Statistical comparisons were performed using t tests, and p values were adjusted for multiple comparisons using false discovery rate. Asterisks indicate >3-fold, statistically significant modulation of gene expression as compared to DMSO control, as follows: * = FDR<0.05; ** = FDR<0.01, and *** = FDR<0.001. (**B**) APOBEC3G expression in isolated CD4+ T cells from HIV-infected ART-suppressed individuals, treated as described in panel **A**. Statistical comparisons were performed using two-tailed Wilcoxon signed-rank tests compared to the DMSO-treated control. **(C-D)** APOBEC3G protein expression in CD4+ T cells treated with either 500 nM rGal-9 or interferon-α (5000 units/ml), as determined by western blot. Immunoblotting bands were quantified with ImageJ software. The quantified APOBEC3G protein expression levels were normalized to corresponding Tubulin protein levels to account for variation in loading.

Achieving reservoir clearance or sustained virologic control of HIV following LRA administration will likely require involvement of innate and intrinsic immunity [8]. We therefore sought to determine the effects of rGal-9 on the expression of genes involved in innate immunity, cell-intrinsic immunity, and cytokine production (S9 Fig). We implemented a qPCR array to measure the expression of 42 established anti-HIV host restriction factors *in vitro* (in J-Lat 5A8 cells) and *ex vivo* (in primary CD4+ T cells isolated from ten ART-suppressed HIV-infected individuals). rGal-9 significantly modulates the expression of several established anti-HIV cell-intrinsic immune defenses. Of particular relevance, we determined that the APOBEC3 cytidine deaminases, including APOBEC3G, were potently induced by rGal-9 *in vitro* (up to 55 fold) (S10 Fig). This induction was confirmed by targeted digital RT-dPCR at the mRNA level (Fig 6A–6C) and by western blotting at the protein level (Fig 6D and 6E). In addition, we determined that APOBEC3G expression was induced by rGal-9 *ex vivo* (p = 0.016) in CD4+ T cells from HIV-infected ART-suppressed individuals at the mRNA level (up to 14 fold) using qPCR (Fig 7A and 7B, S11 Fig) and at the protein level using western blotting (Fig 7C and 7D). In contrast to the induction observed in response to rGal-9 treatment, APOBEC3G expression was significantly reduced by vorinostat treatment *ex vivo* (p = 0.016) (Fig 7A and 7B).

### rGal-9 significantly reduces infectivity of progeny virus

**Fig 8:**
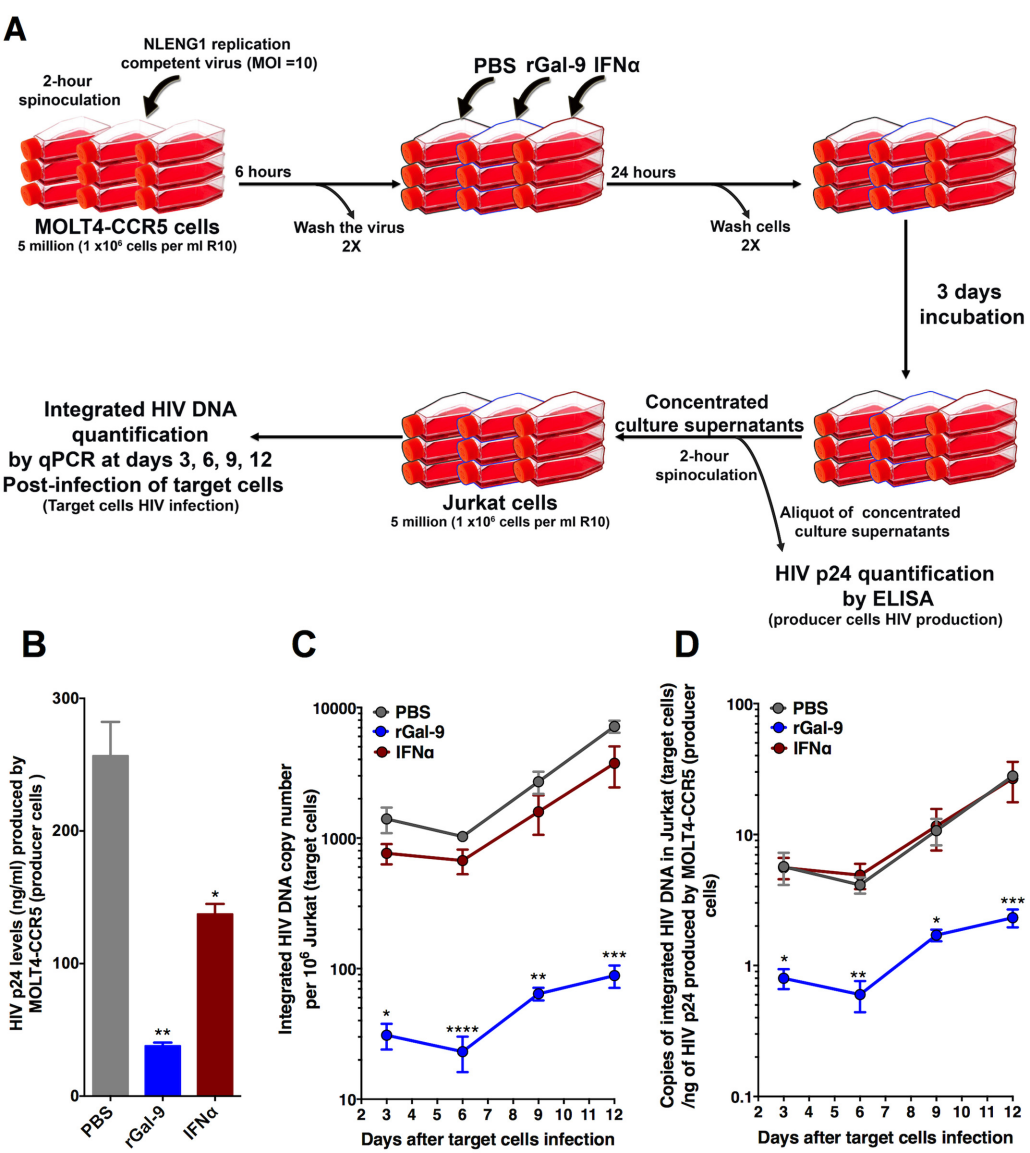
rGal-9 treatment reduces viral infectivity. (**A**) Illustrative schematic of the viral infectivity experiment. (**B-D**) Effects of rGal-9 treatment of producer cells on HIV infectivity. The MOLT4-CCR5 cell line was infected for 6 hours, cells were washed and treated with either PBS, rGal-9 200nM, or interferon-α (5000 U/ml) for 24 hours, and cultures were incubated for 3 days. (**B**) HIV p24 levels produced by MOLT4-CCR5 cells were quantified after concentrating the culture supernatants. Concentrated culture supernatants were used to infect Jurkat cells by spinoculation. (**C**) Levels of integrated HIV DNA measured at days 3, 6, 9, and 12 post-infection of Jurkat cells. (**D**) Levels of integrated HIV DNA at days 3, 6, 9, and 12 post-infection of Jurkat cells, normalized to producer cell p24 supernatant levels. Mean ± SEM is displayed, and statistical comparisons were performed using two-tailed unpaired t test. * = p<0.05; ** = p<0.01, *** = p<0.001, and **** = p<0.0001.

APOBEC3G is an innate antiviral factor that is incorporated into virions and drives extensive G-to-A mutation, or “hypermutation”, of the HIV genome, typically rendering it non-viable within a single replicative cycle [52,53]. We therefore hypothesized that latent virus that is reactivated by rGal-9 (in the setting of elevated APOBEC3G expression) will likely be hypermutated and rendered replication incompetent upon infection of a new cell. To test the hypothesis that virus produced in the presence of rGal-9 is associated with reduced infectivity, we spinoculated MOLT4-CCR5 cells with replication-competent HIV for two hours. After six hours of incubation, cells were washed and were treated with PBS, 200 nM of rGal-9, or 5000U/ml of interferon-α for 24 hours, followed by incubation for three days (Fig 8A). Culture supernatants were concentrated and aliquots of the concentrated culture supernatants were used to measure HIV production by quantifying viral p24 antigen concentration (Fig 8B), and equal amounts of the remaining culture supernatants were used to infect Jurkat cells (by spinoculation) for 12 days. Alu-qPCR was used to quantify the levels of integrated HIV in the Jurkat target cells every 3 days (Fig 8A). rGal-9 treatment of producer cells resulted in a 7.1-fold reduction in the level of integrated HIV DNA in target cells (normalized to producer cell p24 supernatant levels) after 3 days of culture. This reduction remained stable up to 12 days (6.9 fold at day 6, 6.3 fold at day 9, and 12.1 fold at day 12) (Fig 8C and 8D). These data suggest that a significant fraction of the viral particles produced by MOLT4-CCR5 cells treated with rGal-9 were defective in a manner that prevented successful integration into the host genome.

### Soluble galectin-9 (sGal-9) levels in plasma of HIV-infected ART-suppressed individuals are associated with HIV transcription *in vivo*

**Fig 9:**
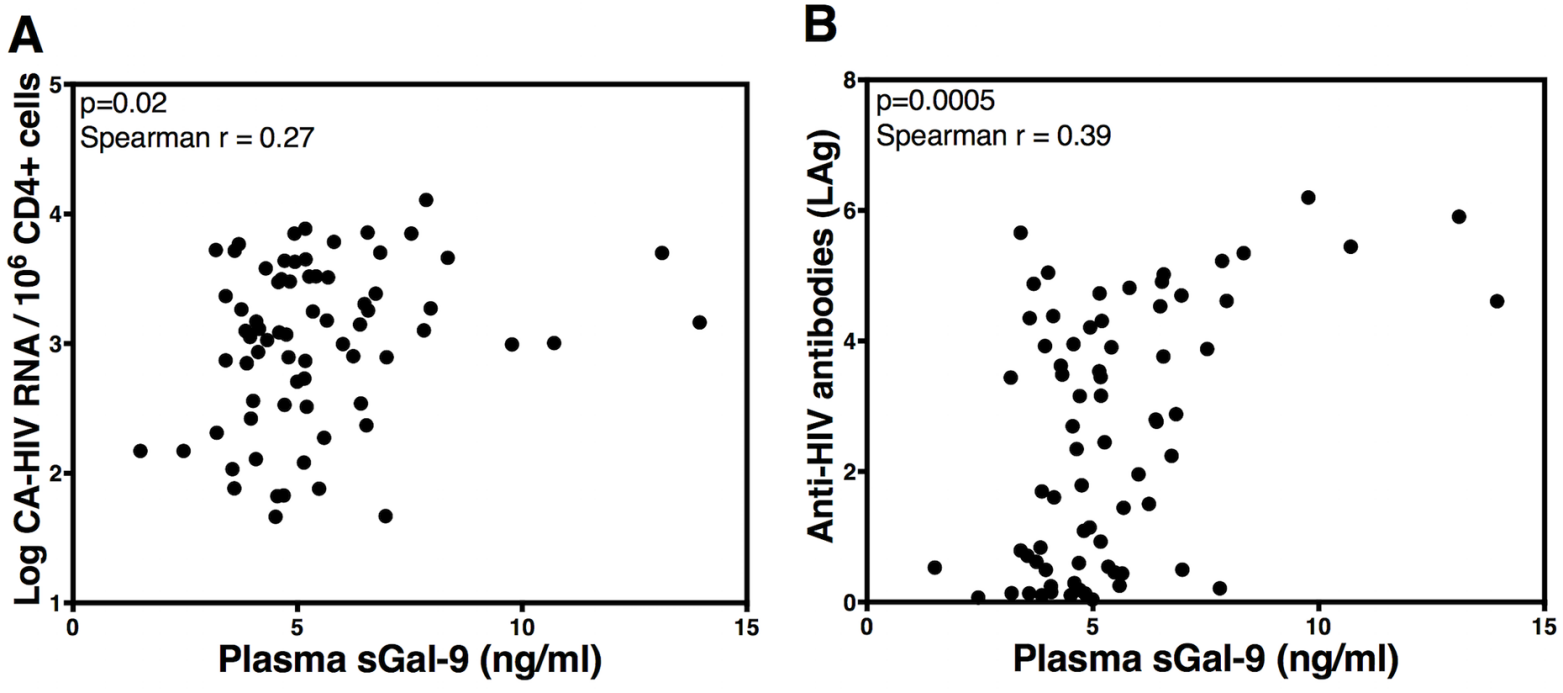
sGal-9 levels correlate with measures of HIV transcription and viral production *in vivo*. Correlations between levels of soluble Gal-9 and (**A**) levels of HIV cell-associated RNA, and (**B**) anti-HIV-1 antibodies in the plasma of 72 HIV-infected ART-suppressed individuals. Correlations were evaluated using Spearman's rank correlation coefficient tests.

Based on our data that rGal-9 potently induces HIV transcription *in vitro* and *ex vivo*, we hypothesized that endogenous sGal-9 regulates HIV transcription *in vivo*, in HIV-infected, ART-suppressed individuals. We examined relationships between plasma levels of sGal-9 in 72 HIV-infected individuals on suppressive ART and levels of CD4+ T cell-associated HIV RNA (marker of HIV transcription), and the quantity and binding avidity of HIV-specific antibodies, which have been associated with HIV production in multiple studies [4,54,55]. Levels of sGal-9 were positively correlated with levels of CD4+ T cell-associated HIV RNA (p = 0.02, Spearman r = 0.27), as well as with the quantity and binding avidity of anti-HIV antibodies (p = 0.0005, Spearman r = 0.39) (Fig 9A and 9B). These data suggest that endogenous Gal-9 likely plays an important role in regulating HIV transcription and viral production during suppressive therapy.

## Discussion

In this study, we have revealed a novel biological function of lectins and cell surface carbohydrates in the biology of viral latency. Our findings strongly suggest that manipulation of Gal-9 (via modulation of endogenous production or exogenous administration of recombinant protein) should be explored as a foundation for novel HIV curative strategies. A large body of literature demonstrates that recombinant Gal-9 is well-tolerated and exhibits therapeutic potential for a range of disorders in animal models [27–31], reinforcing its potential as a therapeutic tool for HIV infection. Furthermore, the observed synergy with the bromodomain inhibitor JQ1 suggests that Gal-9 may serve as a useful component of multi-drug HIV eradication cocktails.

Galectins recognize galactose-containing saccharide sequences displayed on cell surface glycoconjugates [21]. Activated CD4+ T cells and HIV latently-infected cells exhibit an altered cell surface glycosylation pattern with respect to resting, uninfected cells [56]. This aberrant pattern includes loss of sialic acid and an increase of core 2 O-Glycans [56]. In addition, the association between hyposialylation and HIV infection has been established *in vivo* [57,58]. It has been further demonstrated that the loss of sialic acid promotes oligosaccharide binding to Gal-9 and promotes Gal-9 activity [59]. Collectively, these data suggest that the effects of Gal-9 may be more pronounced in HIV latently-infected cells than in uninfected cells. This specificity may allow for potent viral reactivation and reservoir clearance *in vivo* with minimal undesirable perturbation of uninfected cells. Additional studies are needed to decipher the nature of glycan-mediated recognition responsible for Gal-9-mediated signal transduction.

The APOBEC3 cytidine deaminases, including APOBEC3G and APOBEC3F, were strongly induced by rGal-9 in CD4+ T cells. Based on the established mechanism of action associated with the APOBEC3 factors, the pronounced APOBEC3 induction by rGal-9 would be expected to override the antagonistic activity of the HIV Vif protein [60,61] and result in hypermutation of progeny virus upon infection of a new cell, rendering the virus replication incompetent. This phenomenon is supported by our observation that rGal-9 treatment reduces viral infectivity by over seven-fold. This detail is highly relevant to the shock-and-kill HIV cure framework, as it is becoming increasingly clear that ART does not completely block viral replication, especially in tissues with suboptimal drug penetration [62]. APOBEC3 induction in the producer cell will reduce the probability that the HIV reservoir is replenished when latency is reversed therapeutically. This pattern is in stark contrast to our observation that vorinostat significantly suppresses expression of APOBEC3G in CD4+ T cells. This unintended effect of vorinostat may contribute to the published observation that vorinostat enhances cellular susceptibility to HIV infection [63].

The involvement of host immunity will be critical in achieving clearance of the latent HIV reservoir. On this front, rGal-9 was previously found to have immunopotentiating effects [64]. rGal-9 enhanced cytotoxic T cell activity against tumor cells, enhanced IFN-γ and IL-4 production, and promoted dendritic cell maturation via Gal-9-Tim-3 interactions. Our CFSE staining data demonstrate that rGal-9 induces T cell proliferation, compatible with immunopotentiating activity. Although induction of proliferation by an LRA may inadvertently expand the latent reservoir, it is important to note that proliferation driven by rGal-9 was observed primarily in the naïve CD4+ T cell compartment rather than in memory CD4+ T cells. Multiple recent studies have shown that naïve CD4+ T cells rarely harbor latent HIV[65]. Therefore, the induction of CD4+ T cell proliferation by rGal-9 is unlikely to significantly contribute to the expansion of the latent pool. Other studies have demonstrated immunosuppressive effects of rGal-9 in the setting of autoimmune arthritis and organ transplantation rejection models [66,67]. Taken together, it is likely that rGal-9 exhibits immunopotentiating activity in the setting of immunosuppression, and immunosuppressive activity in the setting of hyperimmunity. The effect of rGal-9 on cell-mediated immunity against HIV warrants proper investigation in animal models and clinical studies. Although *in vitro* methods are available to investigate the effects of rGal-9 on innate and adaptive immune responses, recent reports demonstrate that the effects of LRAs on anti-HIV immunity *in vitro* do not accurately reflect the immunologic consequences of administration *in vivo* [13,68].

In summary, our study provides the first evidence that Gal-9 should be considered as a foundation for novel lectin-based strategies to eliminate the latent HIV reservoir. Detailed exploration of biochemical pathways associated with Gal-9 and implementation of animal models will be critical in translating our findings into novel therapeutic or curative approaches for HIV infection.

## Materials and Methods

### Ethics statement

This study utilized retrospectively and prospectively collected specimens from HIV-infected individuals enrolled in the UCSF SCOPE and Hawaii HIV cohorts. Research protocols were approved by the relevant University of California, San Francisco and University of Hawaii Committees on Human Research. All participants were enrolled after obtaining written informed consent, and all subject data and specimens were coded to protect confidentiality.

### Subjects and specimen processing

One milliliter (ml) of plasma was collected retrospectively from 72 ART-suppressed HIV-infected individuals enrolled in the University of California, San Francisco (UCSF) SCOPE and Options cohorts who have been on ART for 1–2 years at time of sampling. We previously measured levels of cell-associated HIV RNA in CD4+ T cells from the same 72 individuals [17,69]. We prospectively recruited 10 participants from the SCOPE cohort. Participants met strict selection criteria and had well-documented persistent viral suppression for over 3 years. This study was approved by the UCSF Committee on Human Research. Fresh blood (100 ml) was collected and peripheral blood mononuclear cells (PBMCs) were isolated from whole blood using SepMate (STEMCELL Technologies). CD4+ T cells were isolated from PBMCs using negative selection (EasySep, STEMCELL Technologies). In addition, one billion cryopreserved PBMCs were collected from five ART-suppressed HIV-infected individuals enrolled in the Hawaii HIV-1 cohort (HHC). Subject characteristics for all 15 participants involved in our prospective sample collection are documented in detail in S1 Table. The characteristics of the 72 ART-suppressed HIV-infected individuals samples used to measure soluble levels of Gal-9 are described in detail in our previous publications [17,69]. CD4+ T cells were enriched from the cryopreserved PBMCs by negative selection using the EasySep Human CD4+ T Cell Enrichment Kit (Stemcell Technologies), according to the manufacturer’s instructions.

### Recombinant and natural galectins

Stable forms of recombinant galectins 1 and 9 were obtained through our collaborators at GalPharma Co., Ltd. (Kagawa, Japan). Galectin-1 and galectin-9 are very unstable in nature, hence stable forms with retaining biological activities were used. For the other galectins (-3, -4, -7, and, -8), the natural, stable form was used [70,71].

### Measurement of HIV latency reversal *in vitro*

We performed experiments in the established “J-Lat” model of HIV latency (kindly provided by Drs. Warner Greene and Eric Verdin, Gladstone Institute of Virology and Immunology). J-Lat cells harbor latent, transcriptionally competent HIV provirus that encodes green fluorescent protein (GFP) as an indicator of viral reactivation [34,35]. We performed dose-response experiments by stimulating J-Lat 5A8, 6.3, and 11.1 clones with varying concentrations of rGal-9 for 24 hours. We used beads coated with anti-human αCD3 (10 μg/ml, eBiosciences, clone:OKT3) and αCD28 (5 μg/ml, eBiosciences, clone: CD28.2) to stimulate T cells in a manner that partially mimics stimulation by antigen-presenting cells as positive control. We used PMA/ionomycin (16 nM/500 nM), and TNFα (10 ng/ml) as positive controls. J-Lat cells were also stimulated with varying concentrations of galectins-1, 3, 4, 7, 8 to evaluate the ability of other recombinant galectins to reverse HIV latency. Flow cytometric analysis in a LSR II flow cytometer using the FACSDiva software (Becton Dickinson, Mountain View, CA) was used to assess mean fluorescence intensity of HIV-encoded GFP expression after stimulation. Data were analyzed with FlowJo (TreeStar Inc., Ashland, OR).

### Measurement of HIV latency reversal *ex vivo*

Isolated CD4+ T cells were plated at a density of 1x10^6^ cells per well at a volume of 1 mL Roswell Park Memorial Institute medium (RPMI) containing 20% FBS (R20) in a 6-well flat-bottom plate. The cells were either untreated or treated with DMSO 0.5% as negative control, PMA (Sigma) at 2 nM and Ionomycin (Sigma) at 0.5 μM, vorinostat at 1 μM, and varying concentrations of rGal-9 (500nM, and 1000 nM) for 24 hours. All stimuli solutions were prepared in R20 culture medium or DMSO solvent from stock solutions. The exposure time of cells to compounds was 24 hours. Total RNA was extracted using the AllPrep DNA/RNA/miRNA Universal Kit (Qiagen) with the optional on-column DNase treatment step. Cellular total HIV-1 RNA was quantified with a qPCR TaqMan assay using LTR-specific primers F522-43 (5’ GCC TCA ATA AAG CTT GCC TTG A 3’; HXB2 522–543) and R626-43 (5’ GGG CGC CAC TGC TAG AGA 3’; 626–643) coupled with a FAM-BQ probe (5’ CCA GAG TCA CAC AAC AGA CGG GCA CA 3) on a ViiA7 Real-time PCR System (Applied Biosystems, Inc. Foster City, CA, USA). Cellular total HIV-1 RNA copy number was determined in a reaction volume of 20 μl with 10 μl of 2x TaqMan RNA-to-Ct 1 Step kit (Life Technologies), 4 pmol of each primer, 4 pmol of probe, 0.5 μl reverse transcriptase, and 5 μl of RNA. Cycling conditions were 48°C for 20 min, 95°C for 10 min, then 60 cycles of 95°C for 15 sec and 59°C for 1 min. External quantitation standards were prepared from full length NL4-3 virion RNA. Specimens were assayed with up to 500 ng cellular total RNA in replicate reaction wells and copy number was determined by extrapolation against a 7-point standard curve (1–10,000 copies) performed in triplicate. Nucleic acid input was normalized for cell number using 18S housekeeping gene copy number by qPCR. For cellular 18S RNA qPCR, the reaction volume was 20 μl with 10 μl of 2x TaqMan RNA to Ct 1 Step kit, 1 μl of human 18S Endogenous Control (Life technologies), 0.5 μl reverse transcriptase, and 5 μl of RNA. Cycling conditions were 48°C for 20 min, 95°C for 10 min, then 40 cycles of 95°C for 15 sec and 60°C for 1 min. 18S external standard curves (8 points curve, from 1–100,000 copies) were prepared from RNA extracted from HIV-1-uninfected CD4^+^ T cells.

### Depletion of CD4+ T cells harboring CD69, CD25, and HLA-DR activation markers

CD4+ T cells were isolated from PBMCs of three HIV-infected ART-suppressed individuals using negative selection (EasySep, STEMCELL Technologies). Resting CD4+ T cells were further enriched through depletion of cells expressing CD69, CD25, or HLA-DR surface markers from half of the isolated CD4+ T cells (CD69 MicroBead Kit II, Miltenyi Biotec; CD25 MicroBeads, Miltenyi Biotec; Anti-HLA-DR MicroBeads, Miltenyi Biotec). The remaining half was processed through the exact enrichment produce, except PBS was added instead of the depleting antibodies. Both of these cell populations were treated with 0.5% DMSO (negative control), 500 nM rGal-9, 1000 nM rGal-9 or αCD3/αCD28-conjugated beads. Induction of cell-associated HIV RNA was measured 24 hours post treatment using RT-qPCR.

### Quantitative analysis of synergy of latency reversing agent combinations

CD4+ T cells isolated from five HIV-infected ART-suppressed individuals were treated with 500 nM of rGal-9, 1 μM vorinostat, 40 nM romidepsin, 10 nM bryostatin, 300 nM prostratin, 1 μM JQ1, or 30 nM panobinostat alone or in combination with 500 nM of rGal-9, in addition to αCD3 + αCD28-conjugated beads (Dynal, at 1:1 bead:cell ratio) as a positive control. Fold induction of cell-associated HIV RNA was determined using quantitative real-time PCR 24 hours after treatment. qPCR data were normalized using three independent methods; cell count post-treatment and immediately prior to total RNA extraction, 18S housekeeping gene quantification by qPCR, and RNA mass quantity measured by NanoDrop Spectrophotometer ND-1000 (NanoDrop Technologies). We adapted the Bliss independence model [48] as implemented by Jiang *et al* and Laird *et al* [14,43] to test for synergy when rGal-9 500nM was combined with several latency reversing agents *ex vivo*. For drugs x and y, we used the equation fa_xyP_ = fa_x_ +fa_y_−(fa_x_)(fa_y_), where fa_xyP_ represents the predicted fraction affected by the combination of drug x and drug y given the observed effects of drug x (fa_x_) and drug y (fa_y_) administered individually and fa_xy_,O = the observed effect when x and y were tested together. Calculation of fa_x_ utilized the following approach adapted from the above cited publications: HIV RNA: fa_x_ = (HIV RNA copies with drug x–background copies with DMSO)/ (HIV RNA copies with αCD3-αCD28 stimulation—background copies with DMSO). In cases where one or more experimental drug conditions resulted in RNA expression exceeding the αCD3-αCD28 stimulation, we imputed the highest HIV RNA value in that experiment +1 to represent the denominator for calculation of fa_x_. Δfa_xy_ = fa_xyO_ (the observed fraction affected by the drug combination)—fa_xyP_ (the predicted fraction affected by the drug combination) provides an indication of synergy (Δ fa_xy_ > 0), additive effect (Bliss independence) (Δ fa_xy_ = 0), or antagonism (Δ fa_xy_< 0). Statistical significance was determined using two-tailed paired t-tests.

### Blocking of Tim-3, PDI, or CD44 receptors

J-Lat 5A8 cells were either untreated were treated with one of the following: 30 mM of α-Lactose monohydrate (Sigma), 5 μg/ml rat anti-human TIM-3 monoclonal antibody (R&D systems, Clone # 344801), 5 μg/ml mouse anti-human PDI monoclonal antibody (Millipore, Clone # 1D3), or 5 μg/ml rat anti-human CD44 monoclonal antibody (Thermo-scientific, Clone # Hermes-1). After 30 minutes of incubation, cells were treated with 200nM of rGal-9. αCD3 (10 μg/ml) and αCD28 (5 μg/ml) stimulation was used as a positive control. Glucose and galactose (30 mM) (Sigma) were used to examine the effects of other saccharide derivatives on rGal-9-mediated HIV latency reversal. Mean fluorescence intensity of HIV-encoded GFP expression in the 5A8 cells was assessed using flow cytometry after 24 hours.

### Cell deglycosylation assay

J-Lat 5A8 cells were either left untreated, or treated with 1 μg/mL tunicamycin (Sigma, St. Louis, MO), enzymatic protein deglycosylation mix (Sigma, St. Louis, MO), or individual deglycosylation enzymes for 24 hours. Cells were washed with RPMI media with 20% FBS, then were either left unstimulated or stimulated with rGal-9 (200 nM), PMA/inonmycin (16 nM, 500 nM), or TNFα (10 ng/ml) for 12 hrs, 24 hrs, or 48 hrs. Mean fluorescence intensity of HIV-encoded GFP expression in the 5A8 cells was assessed using flow cytometry after 24 hours.

### RNA-sequencing of CD4+ T cell transcriptome

J-Lat 5A8 cells were either untreated, or treated with 200 nM of rGal-9, αCD3 (10 μg/ml) and αCD28 (5 μg/ml), or a combination of 200 nM rGal-9 + αCD3 and αCD28 for 24 hours. Cells were sorted based on their GFP expression using the BD FACSAria III. Total RNA was extracted using the AllPrep DNA/RNA/miRNA Universal Kit (Qiagen) with the optional on-column DNase treatment step. RNA was quantified using a NanoDrop Spectrophotometer ND-1000 (NanoDrop Technologies) and integrity was assessed using a 2100 Bioanalyzer (Agilent Technologies). cDNAs were generated using the Illumina TruSeq Stranded mRNA Sample Preparation kit (Illumina Technologies) using 400 ng of total RNA as input. Paired-end sequencing was performed using the Illumina HiSeq 2000 instrument to obtain 30–50 million 2×51 bp reads. RNA-Seq data were preprocessed by adaptor trimming and low quality 3'-tail trimming (Phred > 20). The preprocessed reads were mapped using Tophat[72] to the reference genome hg19. Gene level expression quantification in FPKM (Fragments Per Kilobase of transcript per Million mapped reads) was calculated using Cufflinks suite including Cufflinks, Cuffmerge, Cuffquant and Cuffnorm[72]. Significant changes in transcript expression were quantified using a fold change cutoff (> 2 fold) and t tests to determine significance, adjusted for false discovery rate (FDR <0.05). The gene annotations and gene ontology terms were extracted from BioMart using the Bioconductor/biomaRt package [72].

### CD4+ T cell activation assay

The surface expression of CD69 and CD25 markers of T-cell activation were measured using flow cytometry. Isolated CD4+ T cells were either treated with DMSO 0.5% as negative control, PMA (2 nM) and Ionomycin (0.5 mM), vorinostat (1μg/ml), or two concentrations of rGal-9 (500nM, and 1μM) for 24 hours. Cells were stained with LIVE/DEAD Fixable Aqua Dead Cell Stain Kit (Invitrogen) and then stained with the following fluorescently-conjugated monoclonal antibodies: V450 mouse anti-human CD69 (BD Biosciences, clone: FN50) and APC-cy7 mouse anti-human CD25 (BD Biosciences, clone: M-A251). Rainbow beads (Spherotec) were used to standardize instrument settings between runs. Data were analyzed using FlowJo (TreeStar Inc., Ashland, OR).

### Proliferation assays

CD4+ T cells were negatively selected from thawed cryopreserved PBMCs. Isolated CD4+ T cells were stained with carboxyfluorescein diacetate succinimidyl ester (CFSE) at a final concentration of 10 μM for 15 min. 1 X 10^6^ CFSE-stained CD4+ T cells were either left unstimulated, or stimulated for 24 hours with rGal-9 (200nM, and 500nM). After 24 hours, cells were washed and cultured for another 4 days. Cells were stained with LIVE/DEAD Fixable Aqua Dead Cell Stain Kit (Invitrogen) and then stained with the following fluorescently-conjugated monoclonal antibodies: Brilliant violet 421 anti-human CD4 (BioLegend, clone: OKT4) and FE/cy7 antihuman CD45RA (BioLegend, clone: HI100). Rainbow beads (Spherotec) were used to standardize instrument settings between runs. Data were acquired on the flow cytometer as above and were analyzed with FlowJo (TreeStar Inc., Ashland, OR).

### Quantitative PCR gene expression profiling

400ng of RNA were transcribed into cDNA using random primers and the SuperScript VILO cDNA Synthesis Kit (Invitrogen), according to manufacturer’s instructions. Quantitative real-time PCR utilized custom-made TaqMan Low Density Arrays (TLDA) from Applied Biosystems following the manufacturer’s instructions. Thermal cycling was performed using an Applied Biosystems ViiA 7 Real-Time PCR System. Up to 450 ng cDNA in 200 μl of Applied Biosystems TaqMan Universal PCR Master Mix with UNG was loaded onto the designated ports of the TLDA plates. Data was analyzed using the Applied Biosystems ViiA 7 software. A panel of 6 housekeeping genes was included in the TLDA plates (GAPDH, 18S, ACTB, PPIA, RPLP0, and UBC). 18S ribosomal RNA was identified as the most stably expressed gene from this panel among all samples using the GeNorm algorithm[73]. Therefore, raw cycle threshold numbers of amplified gene products were normalized to 18S ribosomal RNA to control for cDNA input amounts. Fold induction was determined using the comparative Ct method [73].

### Droplet digital PCR gene expression profiling

Starting with 10 ng of input RNA, absolute quantification of HIV *gag*, p21 (Life technologies), and APOBEC3G (Life technologies) mRNA was performed, in addition to RNAse P (Ribonuclease P) as a housekeeping gene, in four-plex digital droplet PCR reactions using the RainDrop system (RainDance Technologies). The RainDrop Source instrument was used with a microfluidic chip (RainDrop Source chip) containing 8 sample wells to generate a collection of uniformly sized (5 picoliter) aqueous droplets from each sample mixed with assay reagents in a reaction volume of 25 μl with 12.5 μl of SuperScript III, 1 μL of SuperScript III Taq Polymerase (Life Technologies), 2.5 μL 10x Droplet Stabilizer (RainDance Technologies), 2.5 μl of each primer/probe mixes, and 5 μl of RNA. Droplets were thermocycled at 50°C for 25 min, 95°C for 10 min, then underwent 45 cycles of 95°C for 15 sec and 60°C for 1 min. The thermal-cycled 8-tube strip was placed into the deck of the RainDrop Sense instrument with a second microfluidic chip (RainDrop Sense chip) used for single droplet fluorescence measurements. Data were analyzed using the RainDrop Analyst software. Data from each sample or control was converted to a 2-dimensional histogram displaying FAM intensity on the x-axis and VIC intensity on the y-axis. Spectral compensation factors were calculated from the positive control data and applied to all samples. Gates used to count the number of droplet events with specific fluorescence properties were defined using graphical tools to outline regions from the positive control, and these gates were applied to all samples. For each sample, the number of PCR-positive droplet events was counted within each gate. Droplet counts were normalized to RNAseP counts.

### Western blotting

Cells were lysed in radioimmunoprecipitation assay buffer (150 mm NaCl, 1% Nonidet P-40 (vol/vol), 0.5% AB-deoxycholate (vol/vol), 0.1% sodium dodecyl sulfate (SDS) (vol/vol), 50 mm Tris-HCl (pH 8), 1 mm DTT), and EDTA-free Protease Inhibitor (Calbiochem). Cellular lysates were used for SDS-polyacrylamide gel electrophoresis (SDS-PAGE) immunoblotting analysis. The primary antibodies used were anti-APOBEC3G mAb (NIH AIDS Reagent Program, Division of AIDS, NIAID, NIH: Anti-APOBEC3G from Dr. Warner C. Greene [74]), anti-p24 mAb (NIH AIDS Reagent Program, Division of AIDS, NIAID, NIH: Anti-HIV-1 p24 Monoclonal (71–31) from Dr. Susan Zolla-Pazner [75]), anti-Vif mAb (NIH AIDS Reagent Program, Division of AIDS, NIAID, NIH: HIV-1 Vif Monoclonal Antibody (#319)) from Dr. Michael H. Malim [76–78]), and anti-Tubulin (B-Tubulin 926–42211 from LI-COR). The immunoblotting bands were quantified using ImageJ software.

### Infectivity assay and virion quantification by p24 ELISA

Five million MOLT4-CCR5 cells (1x10^6^ cells per ml of R10 media) were spinoculated with NLENG1 replication competent virus (MOI = 10) for 2 hours, cells were incubated for 6 hours at 37°C, then washed twice and were treated with PBS, 200 nM rGal-9, or IFNα (5000U/ml) for 24 hours. Then, cells were washed twice and left in culture for 3 additional days. Culture supernatants were concentrated using Lenti-X Concentrator (Clontech) and aliquots of the concentrated culture supernatants were used to measure HIV production through the quantification of viral p24 antigen by p24 HIV Antigen ELISA (PerkinElmer), according to the manufacturer's instructions. The remaining equal amounts of concentrated culture supernatants were used to infect 5 million Jurkat cells (1x10^6^ cells per ml of R10 media) by spinoculation for two hours. Jurkat cells were collected at days 3, 6, 9, and 12 post-infection, and total genomic DNA was extracted using the AllPrep DNA/RNA/miRNA Universal Kit (Qiagen). qPCR was used to quantify the levels of integrated HIV-1 as described below [79].

### Quantification of integrated HIV DNA by qPCR

Integrated HIV DNA levels were quantified using two-step PCR reactions, as previously described[79]. Integrated HIV-1 DNA was pre-amplified with two Alu primers and a primer specific for the HIV LTR region, in addition to primers specific for the CD3 gene to determine cell count. Nested qPCR was then used to amplify HIV and CD3 sequences from the first round of amplification. Specimens were assayed with up to 500 ng cellular DNA in triplicate reaction wells and copy number was determined by extrapolation against a 5-point standard curve (3–30,000 copies), performed in triplicate using extracted DNA from ACH-2 cells.

### sGal-9 level measurement by ELISA

The levels of sGal-9 in the plasma of 72 ART-suppressed HIV-infected individuals were measured using the solid-phase Human Galectin-9 Quantikine ELISA Kit (R&D systems) according to the manufacturer’s instructions.

### Measurement of anti-HIV antibodies in plasma

The Limiting Antigen-Avidity (LAg) EIA (Sedia Biosciences, Portland, OR) was used to quantify and characterize the HIV-specific antibody profile in 72 HIV-infected individuals on suppressive ART. The single-well plate-based LAg EIA measures the quantity and avidity of HIV-specific IgG to subtype B, E and D recombinant HIV-1 envelope antigens [4,54,55].

### Statistical analysis

The nonparametric Mann-Whitney U test was used for unpaired comparisons, Wilcoxon matched-pairs signed ranked test was used for paired comparisons, and Spearman’s r and Pearson's r tests were used for correlation analyses (the Shapiro-Wilk test was used to determine normality prior to implementation of the Pearson’s r test). All statistical analyses were conducted using GraphPad Prism release 6.0 (GraphPad Software, San Diego, CA, USA) and statistical significance was set at a p-value of 0.05. False discovery rates (FDR) were computed using the Benjamini-Hochberg procedure [80] to adjust for multiple comparisons in the RNA-seq data and qPCR array analysis. The heatmaps were generated using standardized Z-scores, and the clustering dendrogram depicting relatedness between gene expression profiles was generated using hierarchical clustering with complete linkage (created using the R statistical package).

## Supporting Information

S1 Fig rGal-9 reactivates latent HIV *in vitro* in an exposure time-dependent fashion. Effects of 1000 nM rGal-9 pulse treatment for 1 hour, 2 hours, 4 hours, and 6 hours compared to continuous treatment for 24 hours. J-Lat cells were analyzed by flow cytometry after 24 hours of culture to assess HIV-encoded GFP expression. Mean ± SEM is displayed, and statistical comparisons were performed using two-tailed unpaired t test. * = p<0.05; ** = p<0.01, *** = p<0.001, and **** = p<0.0001.
(TIFF)

S2 Fig rGal-9 treatment induces the expression of HIV proteins *in vitro*. **(A)** HIV p24 and Vif protein expression in J-Lat 5A8 cells treated with varying concentrations of rGal-9 (100 nM, 200nM, and 500nM) or interferon-α (5000 units/ml), as determined by western blot. **(B-C)** Immunoblotting bands were quantified with ImageJ software. The quantified HIV p24 **(B)** and Vif **(C)** protein expression levels were normalized to corresponding Tubulin protein levels to account for variation in loading.
(TIFF)

S3 Fig Limited-term exposure to rGal-9 reactivates latent HIV ex vivo in CD69 / CD25 / HLA-DR-depleted CD4+ T cells. Effects of 500 nM or 1000 nM rGal-9 treatment for 6 hours compared to continuous treatment for 24 hours. CD4+ T cells were isolated from PBMCs of three HIV-infected ART-suppressed individuals using negative selection. Resting CD4+ T cells were further enriched through depletion of cells expressing CD69, CD25, or HLA-DR surface markers from half of the isolated CD4+ T cells. Cells were treated with 0.5% DMSO (negative control), 500 nM rGal-9, 1000 nM rGal-9 or αCD3/αCD28-conjugated beads for either 6 hours or 24 hours. Induction of cell-associated HIV RNA was measured 24 hours post treatment using RT-qPCR. Mean ± SEM is displayed. Percentages reported reflect average values measured in the 6 hours treatment with respect to values observed with continuous treatment for 24 hours at the same concentration. Each individual is represented with a different symbol.
(TIFF)

S4 Fig Effects of rGal-9 administration on cell viability *ex vivo*. Percentage of live CD4+ T cells from three ART-suppressed individuals treated with 0.5% DMSO as negative control, 500 nM rGal-9, or 1000 nM rGal-9 for 24 hours. LIVE/DEAD Fixable Aqua Dead Cell Staining was used to assess the cellular viability. Mean ± SEM is displayed.
(TIFF)

S5 Fig Glucose and galactose do not inhibit rGal-9-mediated latent viral reactivation. Effects of αLactose (30mM), glucose (30 mM), and galactose (30 mM) treatment on rGal-9-mediated reactivation of HIV in J-Lat 5A8 cells. J-Lat cells were analyzed by flow cytometry to assess HIV-encoded GFP expression.
(TIFF)

S6 Fig Reactivation of latent HIV by PMA/Ionomycin and TNFα is not reduced by deglycosylation. (**A**), Effects of 1μg/ml tunicamycin treatment on the ability of PMA/Ionomycin and TNFα to reactivate latent HIV in J-Lat 5A8 cells. (**B**) Effects of deglycosylation enzymatic mix treatment on the ability of PMA/Ionomycin and TNFα to reactivate latent HIV in J-Lat 5A8 cells. Mean ± SEM is displayed.
(TIFF)

S7 Fig rGal-9 treatment induces a unique gene expression signature in J-Lat cells. Cluster dendrogram depicting relatedness between individual gene expression profiles of sorted GFP-positive and GFP-negative cells containing reactivated (transcriptionally active) HIV proviruses and latent (transcriptionally inactive) proviruses, respectively, after rGal-9 stimulation, αCD3/αCD28 stimulation, or a combination of both. The dendrogram was generated using hierarchical clustering with complete linkage.
(TIFF)

S8 Fig rGal-9 modulates the expression of selected genes associated with T cell activation, T cell proliferation and T cell apoptosis. Heat maps demonstrate the effects of rGal-9 treatment on the expression of (**A**) T cell activation genes, (**B**) genes involved in positive regulation of T cell proliferation, (**C**) genes involved in negative regulation of T cell proliferation, and (**D**) T cell apoptosis genes. Heat colors show standardized Z-scores across samples; red indicates upregulated expression, and green indicates downregulated expression. Heat maps only show genes modulated >2 fold with FDR<0.05. Gene names in red represent genes that were upregulated in the rGal-9-treated, GFP+ cells as compared to unstimulated control. Gene names in green represent genes that were downregulated in rGal-9-treated, GFP+ cells as compared to unstimulated control. The gene annotations and gene ontology terms were extracted from BioMart using the Bioconductor/biomaRt package.
(TIFF)

S9 Fig rGal-9 modulates the expression of selected genes associated with innate immunity, cytokine production, and interferon signaling. Heat maps demonstrate the effects of rGal-9 treatment on the expression of (**A**) innate immunity-associated genes, (**B**) cytokine genes, and (**C**) interferon genes. Heat colors show standardized Z-scores across samples; red indicates upregulated expression, and green indicates downregulated expression. Heat maps only show genes modulated >2 fold with FDR<0.05. Gene names in red represent genes that were upregulated in the rGal-9-treated, GFP+ cells as compared to unstimulated control. Gene names in green represent genes that were downregulated in rGal-9-treated, GFP+ cells as compared to unstimulated control. The gene annotations and gene ontology terms were extracted from BioMart using the Bioconductor/biomaRt package.
(TIFF)

S10 Fig rGal-9 treatment induces the expression of several anti-HIV host restriction factors including APOBEC3G *in vitro*. Heat map depicts expression levels of host restriction factors in sorted GFP-positive and GFP-negative J-Lat 5A8 cells containing reactivated (transcriptionally active) HIV proviruses and latent (transcriptionally inactive) proviruses, respectively, after rGal-9 stimulation, αCD3/αCD28 stimulation, or a combination of both. Heat colors scale with fold modulation compared to the unstimulated control as described in the figure.
(TIFF)

S11 Fig Modulation of gene expression by rGal-9 is similar *in vitro* and *ex vivo*. (**A**) Correlations between gene expression modulation of 42 anti-HIV-1 host restriction factors in J-Lat 5A8 cells treated with 200nM of rGal-9, and primary CD4+ T cells from 10 HIV-infected, ART-suppressed individuals treated with either 500nM of rGal-9 or 1000nM of rGal-9. (**B**) Correlation between gene expression modulation of 42 anti-HIV-1 host restriction factors in primary CD4+ T cells treated with 500nM of rGal-9, and 1000nM of rGal-9. Correlations were evaluated using Pearson's r tests after testing for normality using the Shapiro-Wilk test.
(TIFF)

S1 Table Subject characteristics. (TIFF)
