# Supplementary figures and images for: Human Galectin-9 Is a Potent Mediator of HIV Transcription and Reactivation
Source: PLoS Pathog. 2016 Jun 2;12(6):e1005677. doi: 10.1371/journal.ppat.1005677 (PMC4890776; doi:10.1371/journal.ppat.1005677)

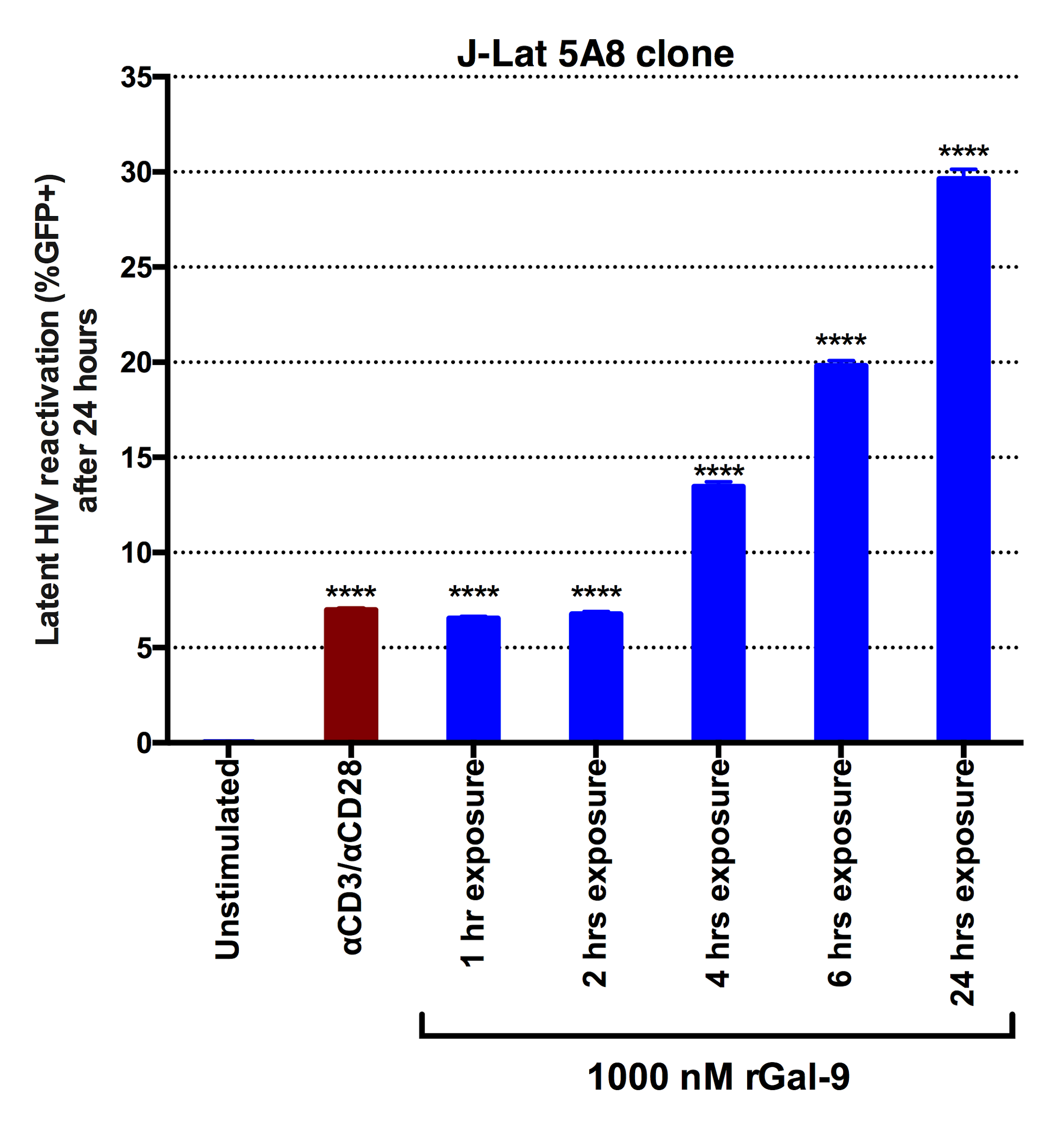

Supplement: S1 Fig — Effects of 1000 nM rGal-9 pulse treatment for 1 hour, 2 hours, 4 hours, and 6 hours compared to continuous treatment for 24 hours. J-Lat cells were analyzed by flow cytometry after 24 hours of culture to assess HIV-encoded GFP expression. Mean ± SEM is displayed, and statistical comparisons were performed using two-tailed unpaired t test. * = p<0.05; ** = p<0.01, *** = p<0.001, and **** = p<0.0001. (TIFF) [file ppat.1005677.s001.tiff]

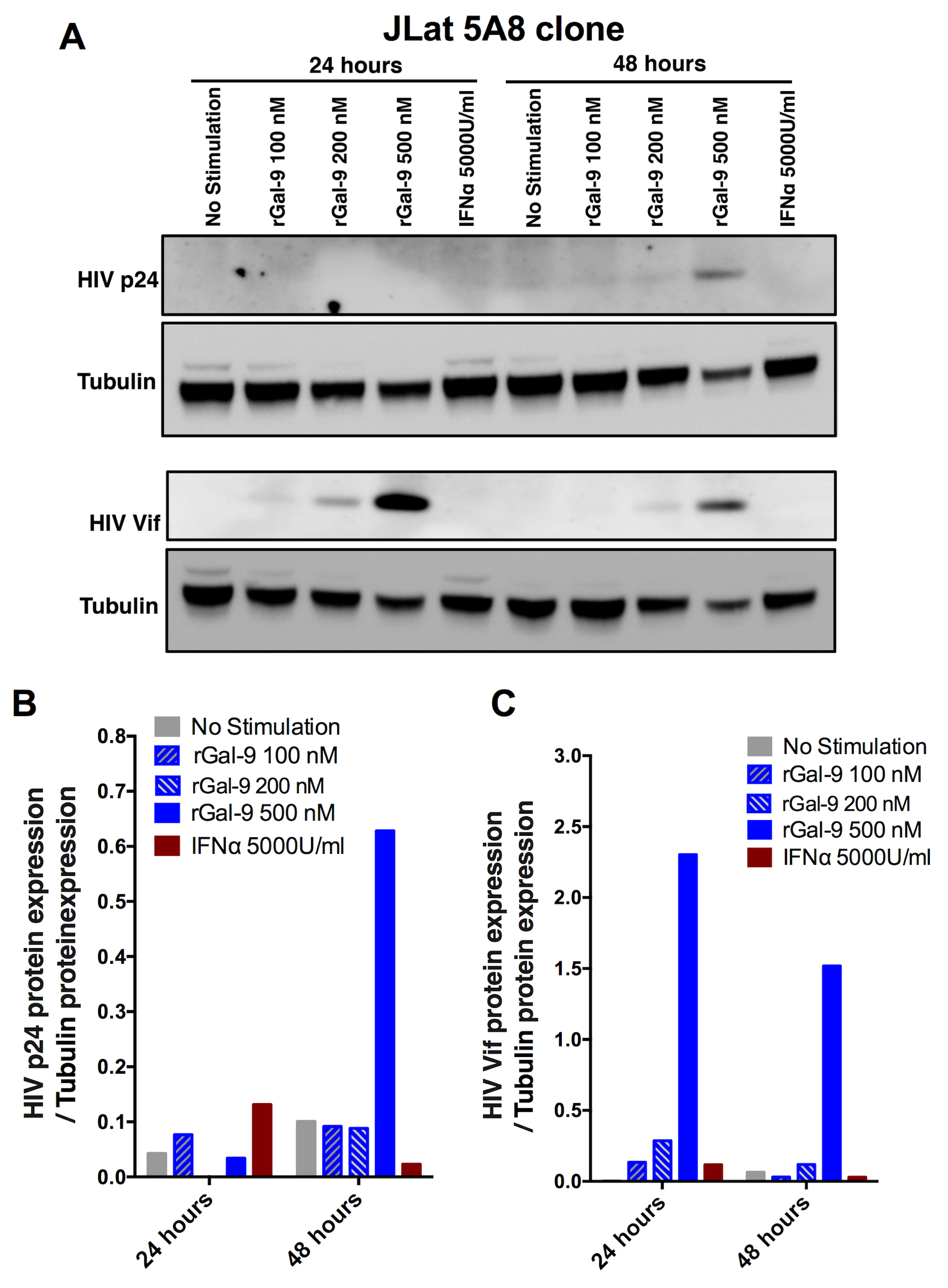

Supplement: S2 Fig — (A) HIV p24 and Vif protein expression in J-Lat 5A8 cells treated with varying concentrations of rGal-9 (100 nM, 200nM, and 500nM) or interferon-α (5000 units/ml), as determined by western blot. (B-C) Immunoblotting bands were quantified with ImageJ software. The quantified HIV p24 (B) and Vif (C) protein expression levels were normalized to corresponding Tubulin protein levels to account for variation in loading. (TIFF) [file ppat.1005677.s002.tiff]

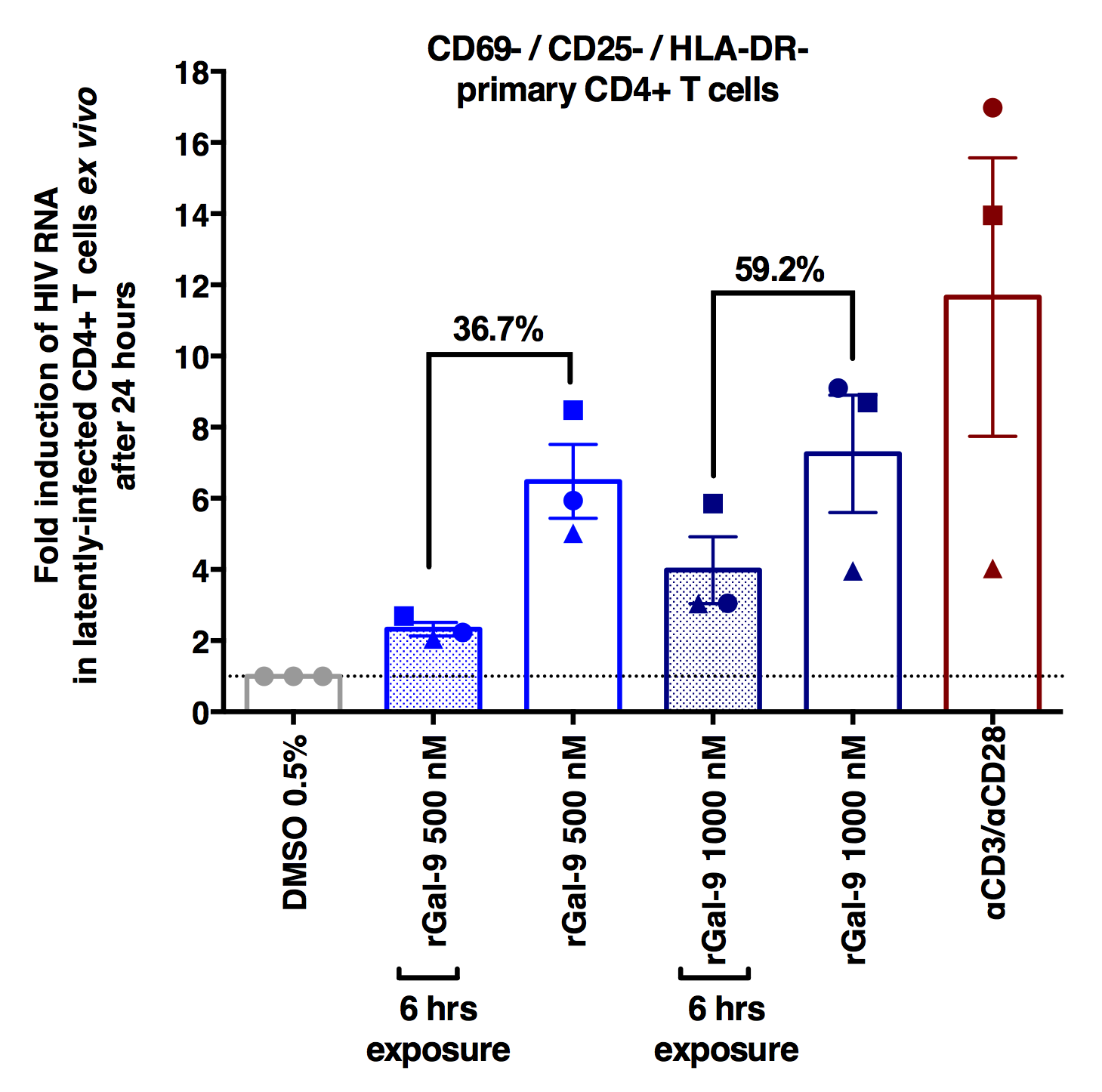

Supplement: S3 Fig — Effects of 500 nM or 1000 nM rGal-9 treatment for 6 hours compared to continuous treatment for 24 hours. CD4+ T cells were isolated from PBMCs of three HIV-infected ART-suppressed individuals using negative selection. Resting CD4+ T cells were further enriched through depletion of cells expressing CD69, CD25, or HLA-DR surface markers from half of the isolated CD4+ T cells. Cells were treated with 0.5% DMSO (negative control), 500 nM rGal-9, 1000 nM rGal-9 or αCD3/αCD28-conjugated beads for either 6 hours or 24 hours. Induction of cell-associated HIV RNA was measured 24 hours post treatment using RT-qPCR. Mean ± SEM is displayed. Percentages reported reflect average values measured in the 6 hours treatment with respect to values observed with continuous treatment for 24 hours at the same concentration. Each individual is represented with a different symbol. (TIFF) [file ppat.1005677.s003.tiff]

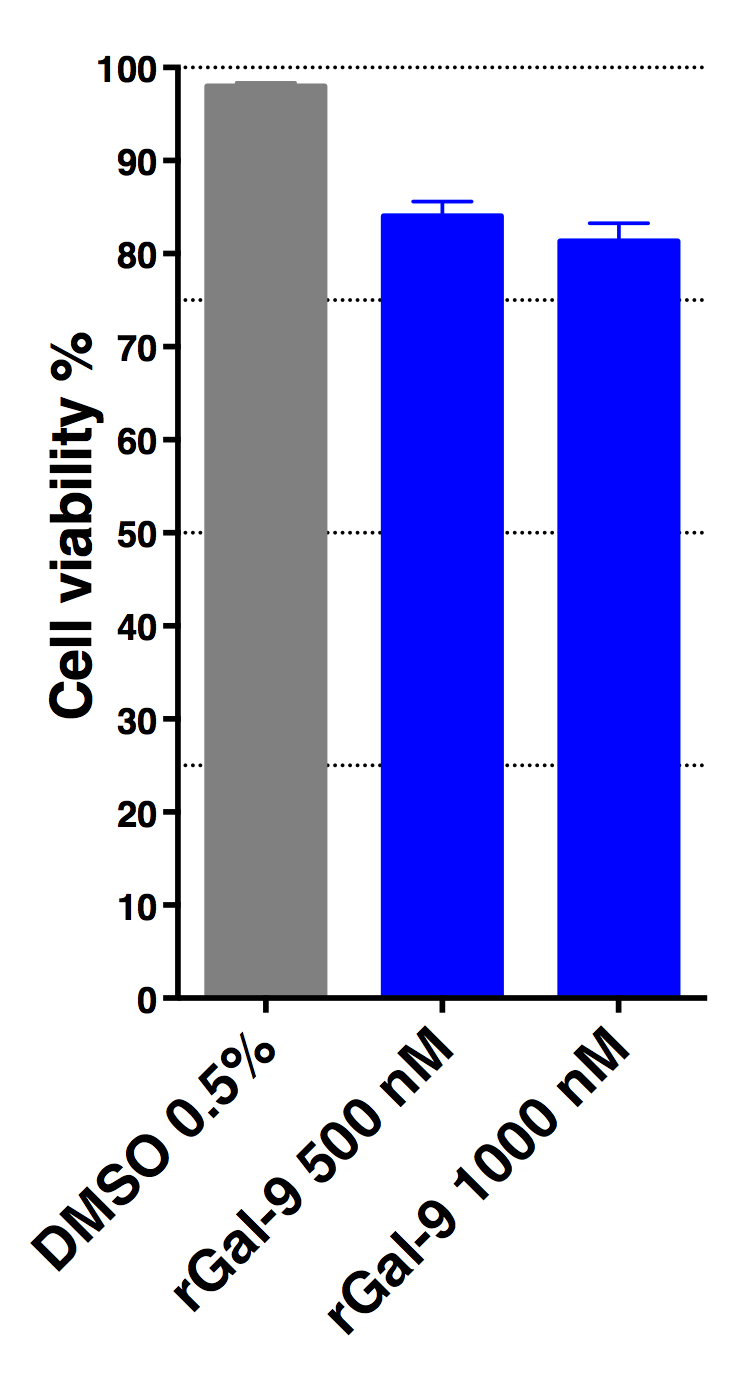

Supplement: S4 Fig — Percentage of live CD4+ T cells from three ART-suppressed individuals treated with 0.5% DMSO as negative control, 500 nM rGal-9, or 1000 nM rGal-9 for 24 hours. LIVE/DEAD Fixable Aqua Dead Cell Staining was used to assess the cellular viability. Mean ± SEM is displayed. (TIFF) [file ppat.1005677.s004.tiff]

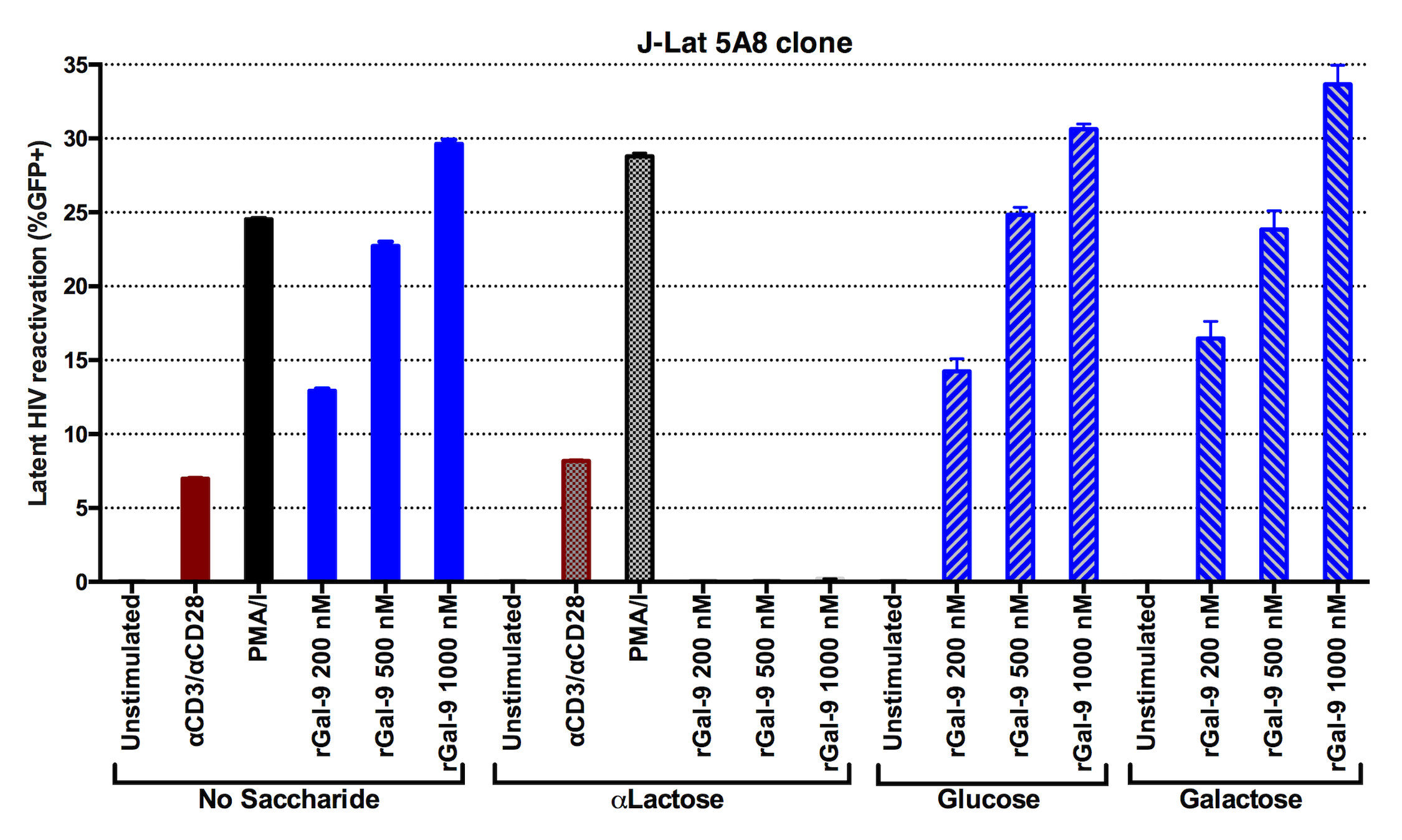

Supplement: S5 Fig — Effects of αLactose (30mM), glucose (30 mM), and galactose (30 mM) treatment on rGal-9-mediated reactivation of HIV in J-Lat 5A8 cells. J-Lat cells were analyzed by flow cytometry to assess HIV-encoded GFP expression. (TIFF) [file ppat.1005677.s005.tiff]

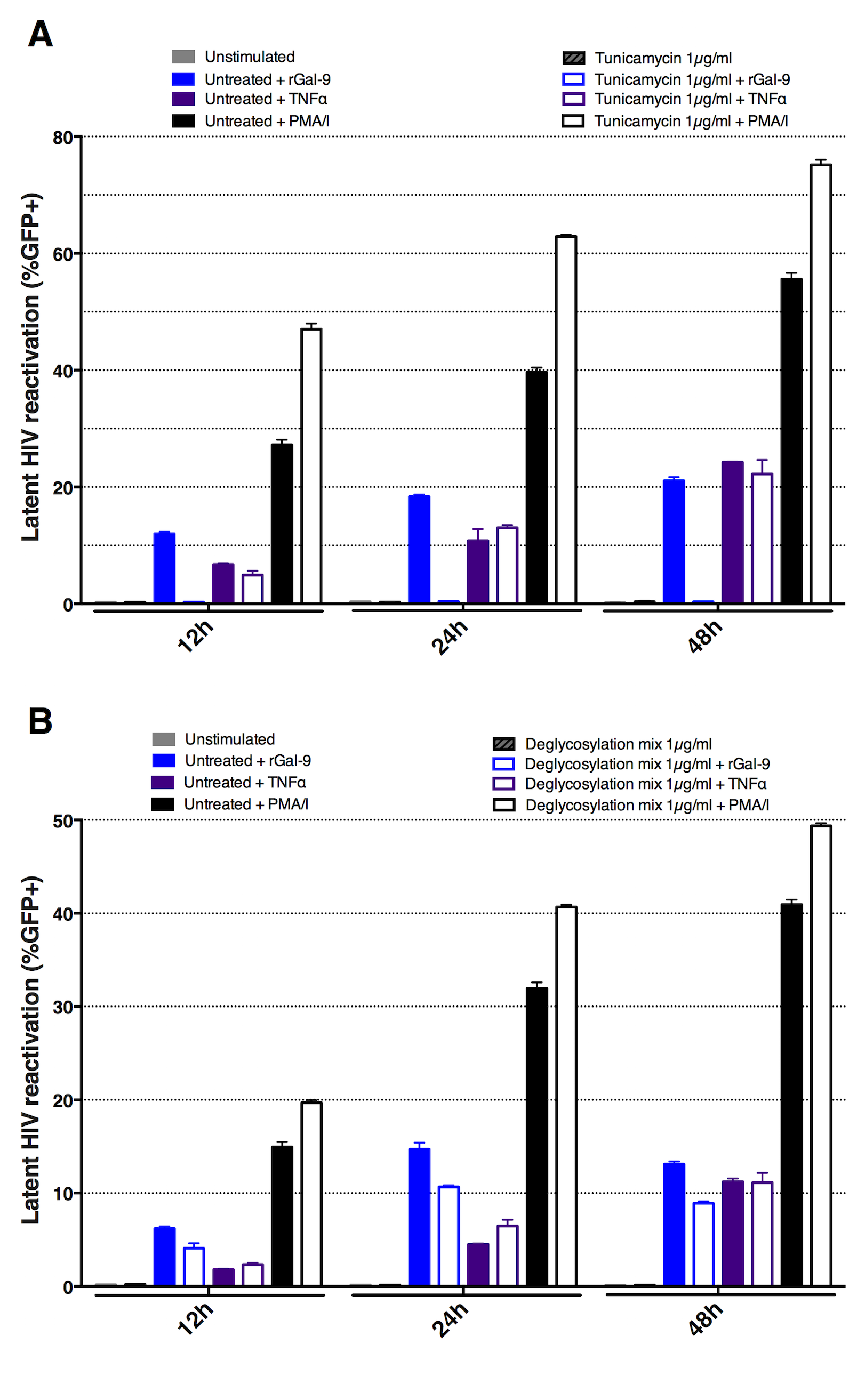

Supplement: S6 Fig — (A), Effects of 1μg/ml tunicamycin treatment on the ability of PMA/Ionomycin and TNFα to reactivate latent HIV in J-Lat 5A8 cells. (B) Effects of deglycosylation enzymatic mix treatment on the ability of PMA/Ionomycin and TNFα to reactivate latent HIV in J-Lat 5A8 cells. Mean ± SEM is displayed. (TIFF) [file ppat.1005677.s006.tiff]

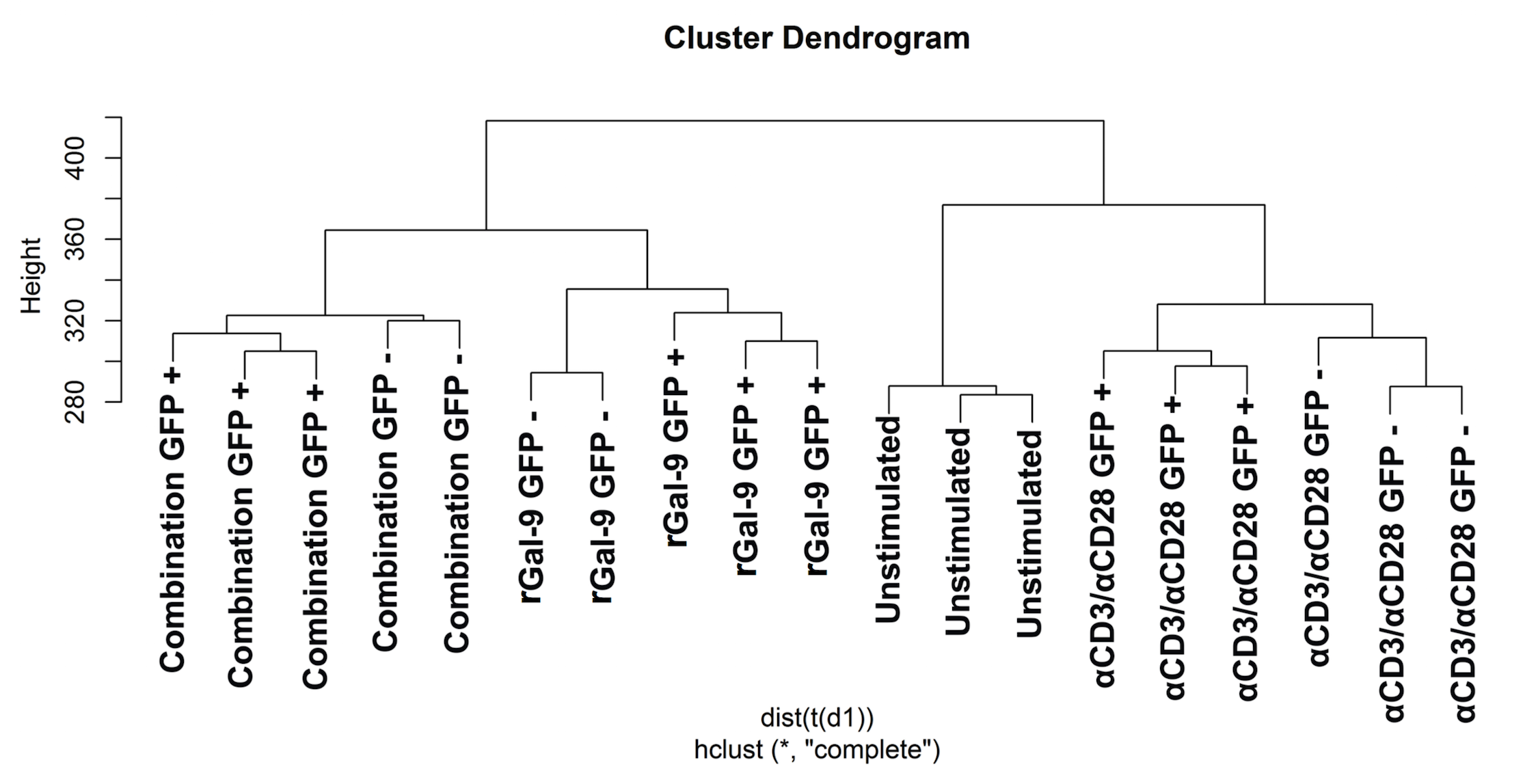

Supplement: S7 Fig — Cluster dendrogram depicting relatedness between individual gene expression profiles of sorted GFP-positive and GFP-negative cells containing reactivated (transcriptionally active) HIV proviruses and latent (transcriptionally inactive) proviruses, respectively, after rGal-9 stimulation, αCD3/αCD28 stimulation, or a combination of both. The dendrogram was generated using hierarchical clustering with complete linkage. (TIFF) [file ppat.1005677.s007.tiff]

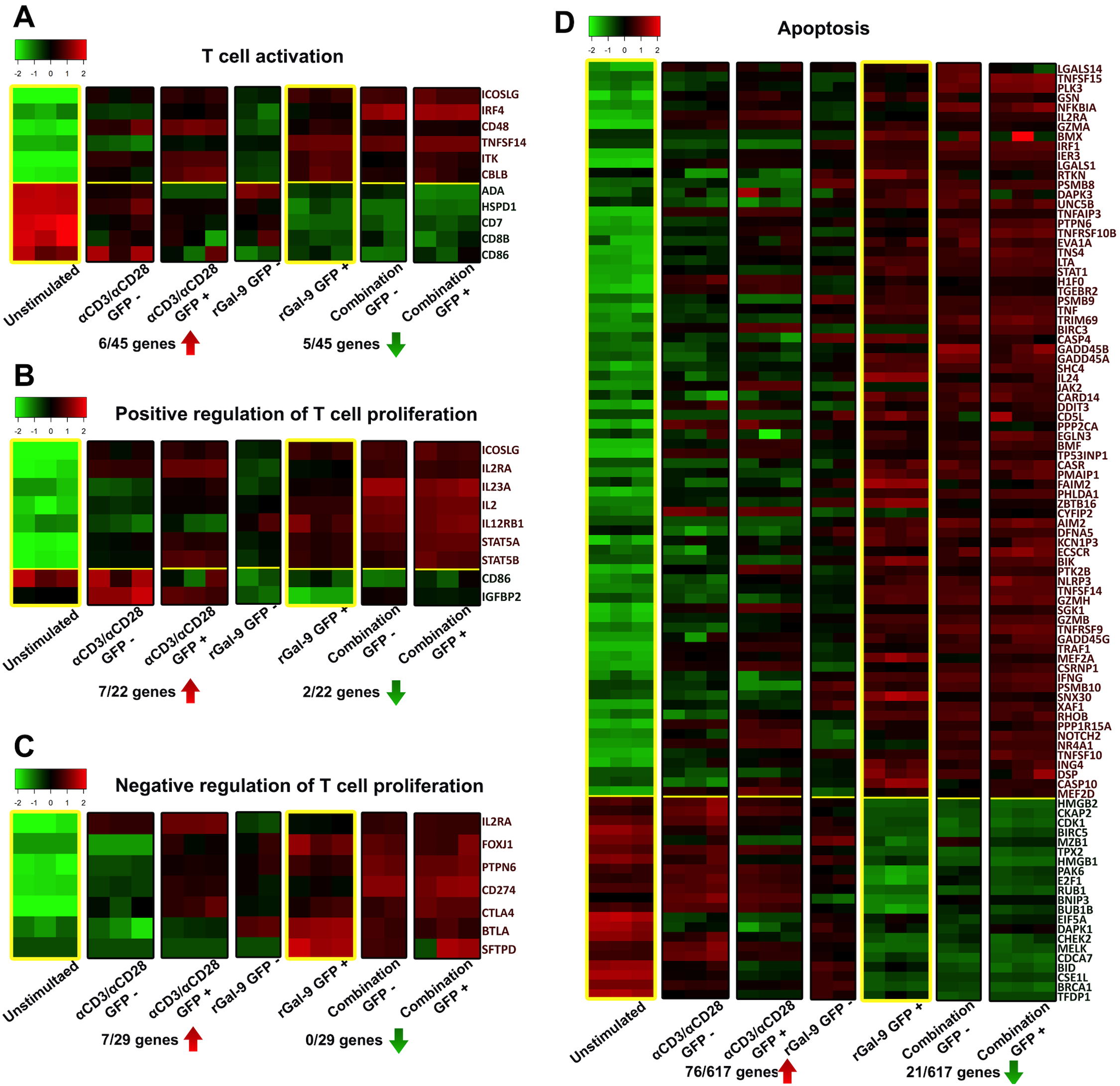

Supplement: S8 Fig — Heat maps demonstrate the effects of rGal-9 treatment on the expression of (A) T cell activation genes, (B) genes involved in positive regulation of T cell proliferation, (C) genes involved in negative regulation of T cell proliferation, and (D) T cell apoptosis genes. Heat colors show standardized Z-scores across samples; red indicates upregulated expression, and green indicates downregulated expression. Heat maps only show genes modulated >2 fold with FDR<0.05. Gene names in red represent genes that were upregulated in the rGal-9-treated, GFP+ cells as compared to unstimulated control. Gene names in green represent genes that were downregulated in rGal-9-treated, GFP+ cells as compared to unstimulated control. The gene annotations and gene ontology terms were extracted from BioMart using the Bioconductor/biomaRt package. (TIFF) [file ppat.1005677.s008.tiff]

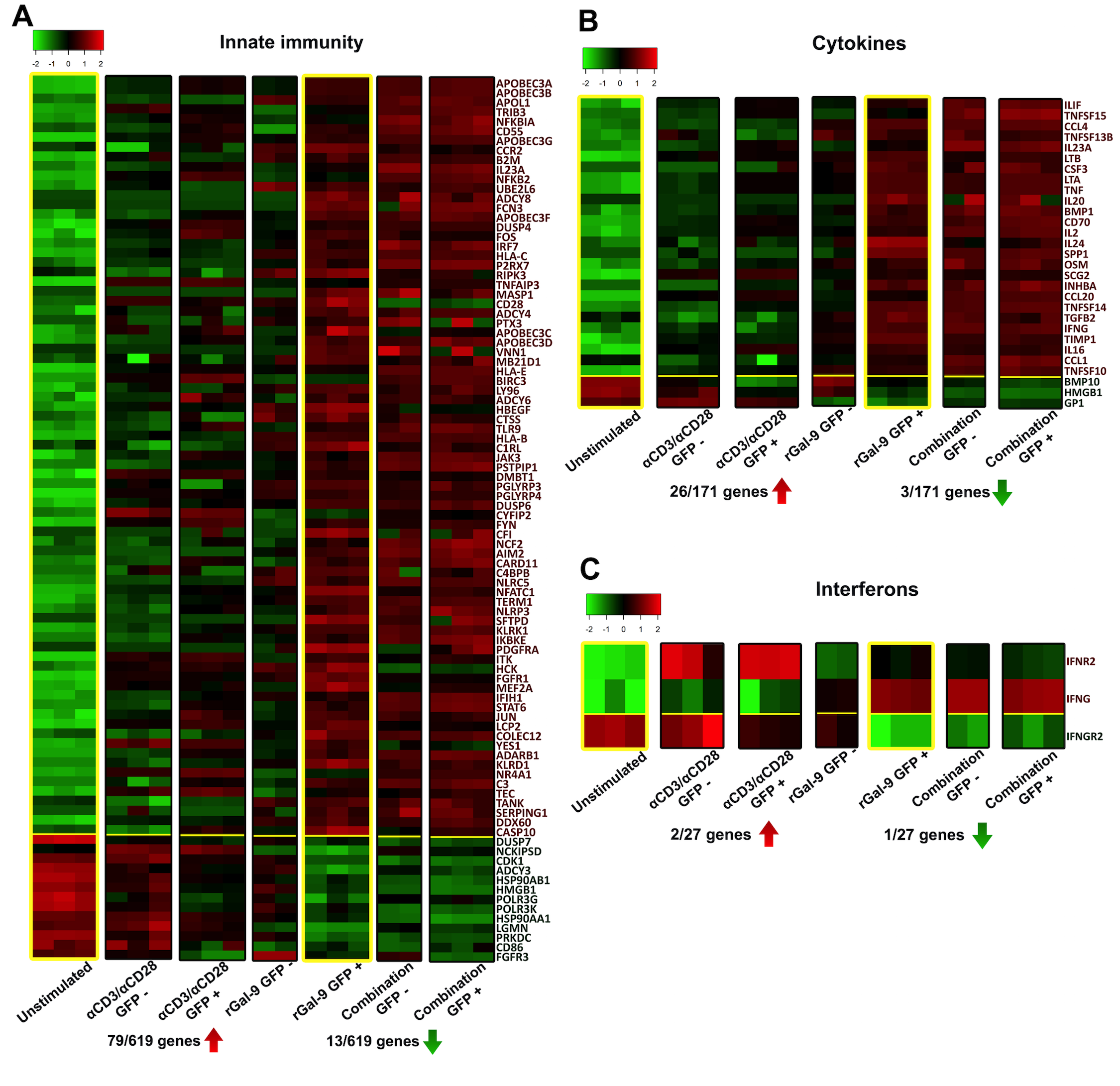

Supplement: S9 Fig — Heat maps demonstrate the effects of rGal-9 treatment on the expression of (A) innate immunity-associated genes, (B) cytokine genes, and (C) interferon genes. Heat colors show standardized Z-scores across samples; red indicates upregulated expression, and green indicates downregulated expression. Heat maps only show genes modulated >2 fold with FDR<0.05. Gene names in red represent genes that were upregulated in the rGal-9-treated, GFP+ cells as compared to unstimulated control. Gene names in green represent genes that were downregulated in rGal-9-treated, GFP+ cells as compared to unstimulated control. The gene annotations and gene ontology terms were extracted from BioMart using the Bioconductor/biomaRt package. (TIFF) [file ppat.1005677.s009.tiff]

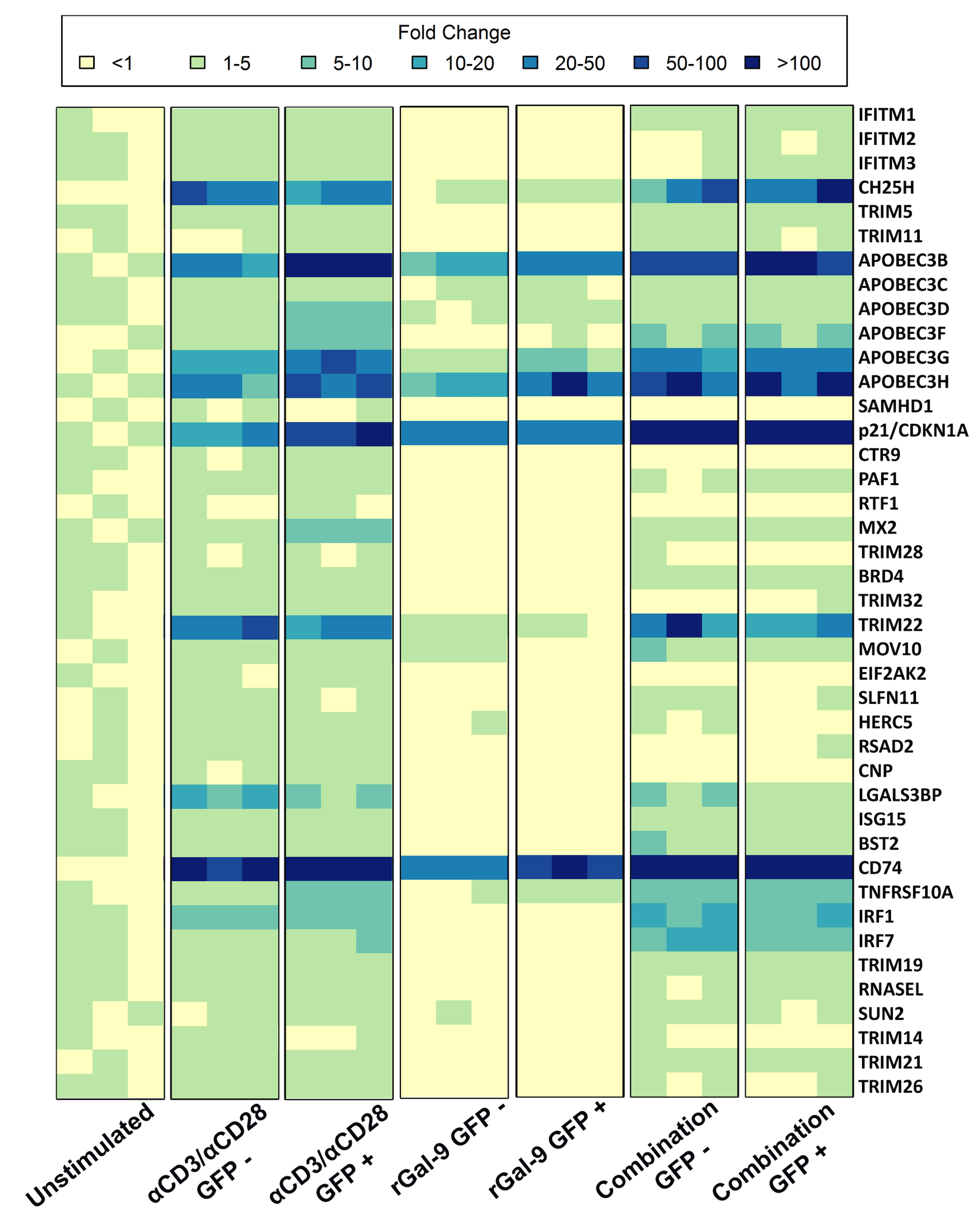

Supplement: S10 Fig — Heat map depicts expression levels of host restriction factors in sorted GFP-positive and GFP-negative J-Lat 5A8 cells containing reactivated (transcriptionally active) HIV proviruses and latent (transcriptionally inactive) proviruses, respectively, after rGal-9 stimulation, αCD3/αCD28 stimulation, or a combination of both. Heat colors scale with fold modulation compared to the unstimulated control as described in the figure. (TIFF) [file ppat.1005677.s010.tiff]

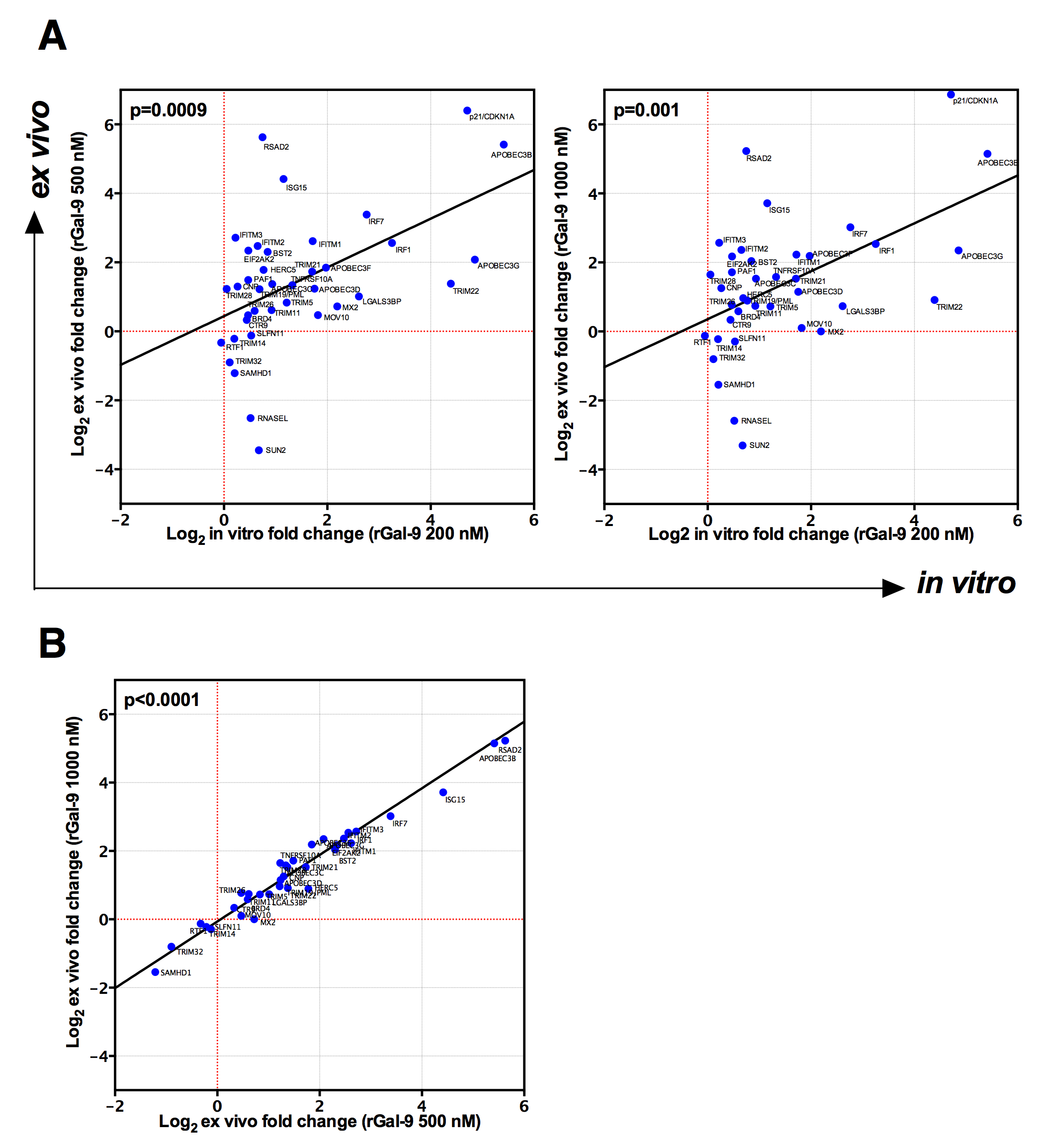

Supplement: S11 Fig — (A) Correlations between gene expression modulation of 42 anti-HIV-1 host restriction factors in J-Lat 5A8 cells treated with 200nM of rGal-9, and primary CD4+ T cells from 10 HIV-infected, ART-suppressed individuals treated with either 500nM of rGal-9 or 1000nM of rGal-9. (B) Correlation between gene expression modulation of 42 anti-HIV-1 host restriction factors in primary CD4+ T cells treated with 500nM of rGal-9, and 1000nM of rGal-9. Correlations were evaluated using Pearson's r tests after testing for normality using the Shapiro-Wilk test. (TIFF) [file ppat.1005677.s011.tiff]

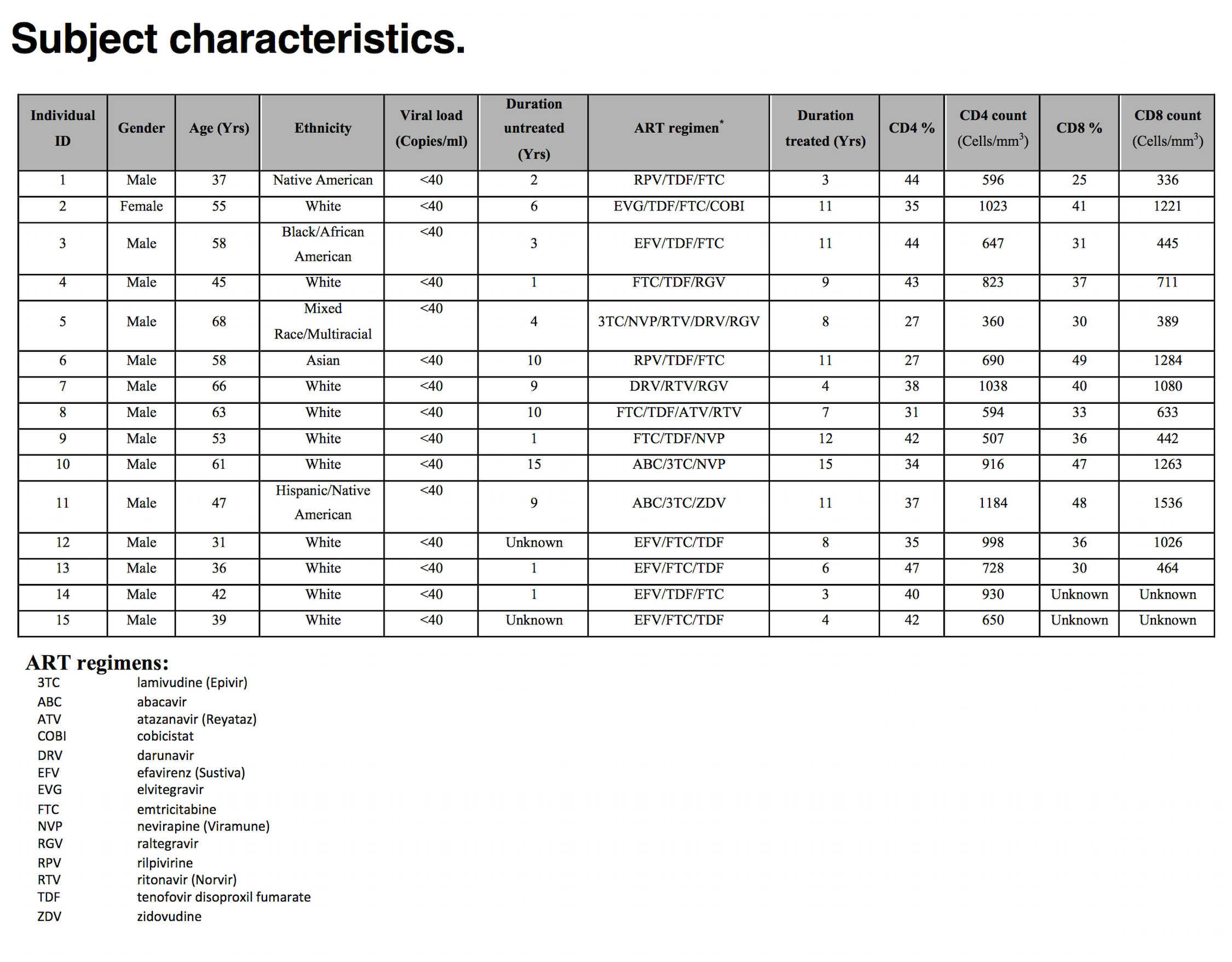

Supplement: S1 Table — (TIFF) [file ppat.1005677.s012.tiff]
